# Magnetically Induced Iron-Catalyzed Hydrodeoxygenation of Benzylic Esters and Polyesters

**DOI:** 10.1021/jacs.5c10464

**Published:** 2025-09-11

**Authors:** Sihana Ahmedi, Lise-Marie Lacroix, Derya Demirbas, Daniel J. SantaLucia, Claudia Weidenthaler, Walid Hetaba, Walter Leitner, Alexis Bordet

**Affiliations:** 1 28313 Max Planck Institute for Chemical Energy Conversion, Stiftstraße 34-36, Mülheim an der Ruhr 45470, Germany; 2 Institute of Technical and Macromolecular Chemistry, RWTH Aachen University, Worringerweg 2, Aachen 52074, Germany; 3 Laboratoire de Physique et Chimie des Nano-Objets, UMR 5215 INSA, CNRS, UPS, Université de Toulouse, 135 Avenue de Rangueil, Toulouse 31077, France; 4 Institut Universitaire de France (IUF), 103 Boulevard Saint Michel, Paris 75005, France; 5 28314 Max-Planck-Institut für Kohlenforschung, Kaiser-Wilhelm-Platz 1, Mülheim an der Ruhr 45470, Germany

## Abstract

The selective hydrodeoxygenation of benzylic esters with molecular hydrogen (H_2_) provides a synthetic approach to methyl-substituted aromatic compounds used widely as intermediates and products in the chemical and pharmaceutical industries in accordance with green chemistry principles. In particular, it can open novel pathways for the use of biomass-derived substrates or waste plastics as chemical feedstocks. We present here an efficient catalytic approach focusing on the use of earth abundant iron in the form of iron carbide nanoparticles (ICNPs) activated by magnetic induction, allowing the reaction to proceed at pressures of only 3 bar of H_2_. The activity of the ICNPs responds in real time to on/off switches of the alternating current magnetic field (ACMF, 350 kHz, 70 mT), mimicking the use of intermittent renewable electricity, and the magnetic properties of the ICNPs allow for their easy separation and reuse. The reaction proceeds with higher yield and selectivities at global temperatures more than 130 °C below thermal activation, leading to at least four times higher energy efficiency. The method was successfully applied to a range of synthetic targets and to the selective depolymerization of real polyester (PET) products.

## Graphical abstract

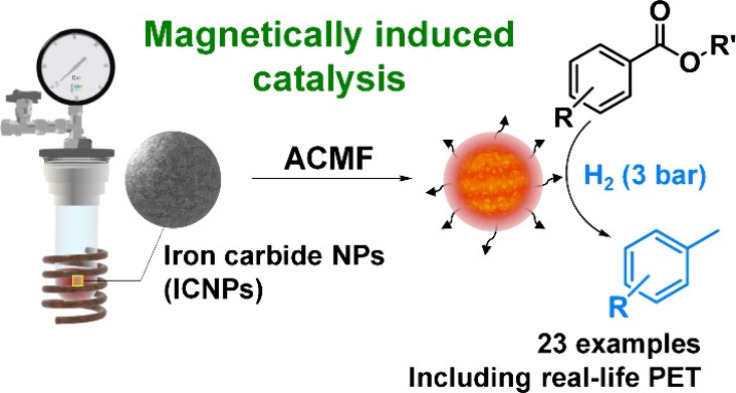

## Introduction

The selective hydrodeoxygenation of benzylic esters to methyl-substituted aromatic compounds such as tolyl derivatives (Figure 1A) is attractive for the synthesis of fine chemicals (e.g., dyes, pigments, etc.) and pharmaceuticals (e.g., analgesics, antihistamines, etc.)^1^ and has the potential to valorize biobased intermediates and waste polyesters into the chemical value chain.^1–3^ However, it is a complex transformation that requires a 6e-reduction and breaking of all C–O bonds at the ester group without affecting the aromatic ring. Current methods for selective hydrodeoxygenation of benzylic esters rely on stoichiometric or even excess amounts of strong and hazardous reducing agents (e.g., LiAlH_4_, NaBH_4_), which pose significant safety concerns and limitations in terms of the atom economy and functional group tolerance.^4,5^ Catalytic approaches are much scarcer with recent developments focusing on hydroboration and hydrosilylation using metal complexes in sophisticated catalytic systems (Figure 1B).^6–9^ Using molecular hydrogen (H_2_) as a reducing agent would be highly desirable in order to comply with the concepts of green and sustainable chemistry. The very few reports using H_2_ or H_2_ sources such as methanol for benzylic ester hydrodeoxygenation require large amounts of copper-based multimetallic heterogeneous catalysts, need to be conducted in stainless-steel high-pressure equipment, remain poorly selective, and have a scope of applications limited to dimethyl terephthalate.^10,11^

**Figure 1:**
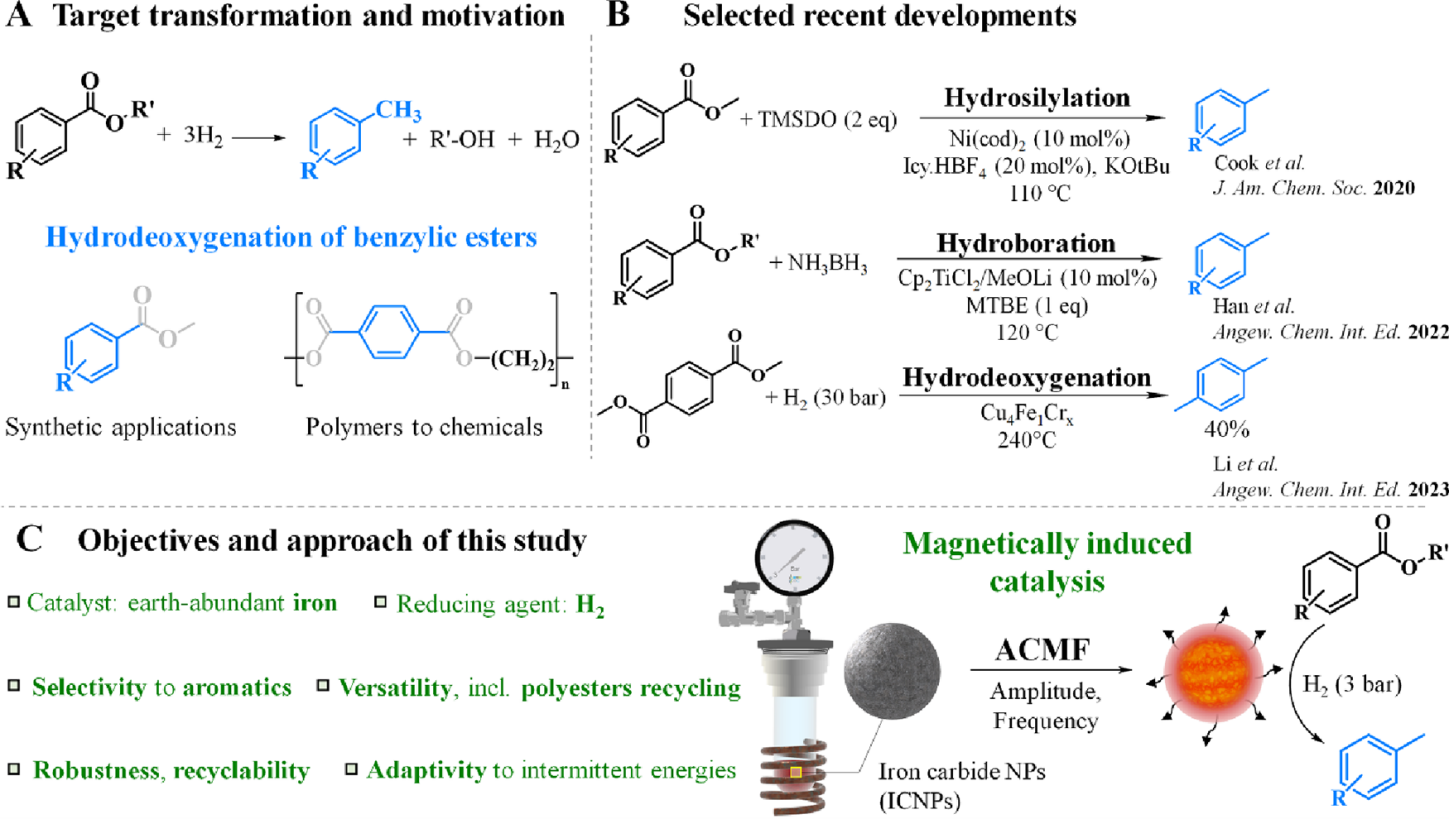
(A) Motivation to study the hydrodeoxygenation of benzylic esters, (B) selected recent examples from the literature, and (C) the objectives and approach of the present study using magnetically induced catalysis.

We demonstrate here that the use of earth-abundant iron in the form of iron carbide nanoparticles (ICNPs) under magnetic induction offers a highly effective catalytic system for hydrodeoxygenation of aromatic esters using molecular hydrogen under comparably mild conditions (Figure 1C). The key to enabling the deep hydrogenation of the ester group to a methyl substituent without affecting the aromatic ring is the activation of the iron-based NPs by magnetic induction using an alternating current magnetic field (ACMF). This area of magnetocatalysis has emerged as a promising approach to enable the selective generation of thermal energy directly at appropriately designed catalysts in an energy efficient, rapid, and localized manner.^12–18^ It can also potentially open new ways to introduce green electricity in catalytic processes while coping with the intermittency of renewable electricity. Applications demonstrated for this method here range from green synthesis to the chemical recycling of polyester waste.

## Results and Discussion

### Catalyst Synthesis and Characterization

Iron carbide nanoparticles (ICNPs) with excellent heating power under ACMF (specific absorption rate (SAR) of ca. 2500 W g^–1^ at *f* = 100 kHz and μ_0_ *H*_max_ = 66 mT) were prepared according to an organometallic approach previously developed by some of us (Figure 2A).^15^ First, {Fe­[N­(SiMe_3_)_2_]_2_}_2_ was reduced under H_2_ in the presence of palmitic acid and hexadecylamine to produce Fe(0) NPs (Figures S1–S2), followed by their carbidizazion under syngas (150 °C in mesitylene, CO/H_2_, 1:1 ratio, 4 bar total pressure) to yield 12.5 ± 1.0 nm ICNPs (Figure 2B, Fe_2.2_C@Fe_5_C_2_ core@shell structure).

**Figure 2:**
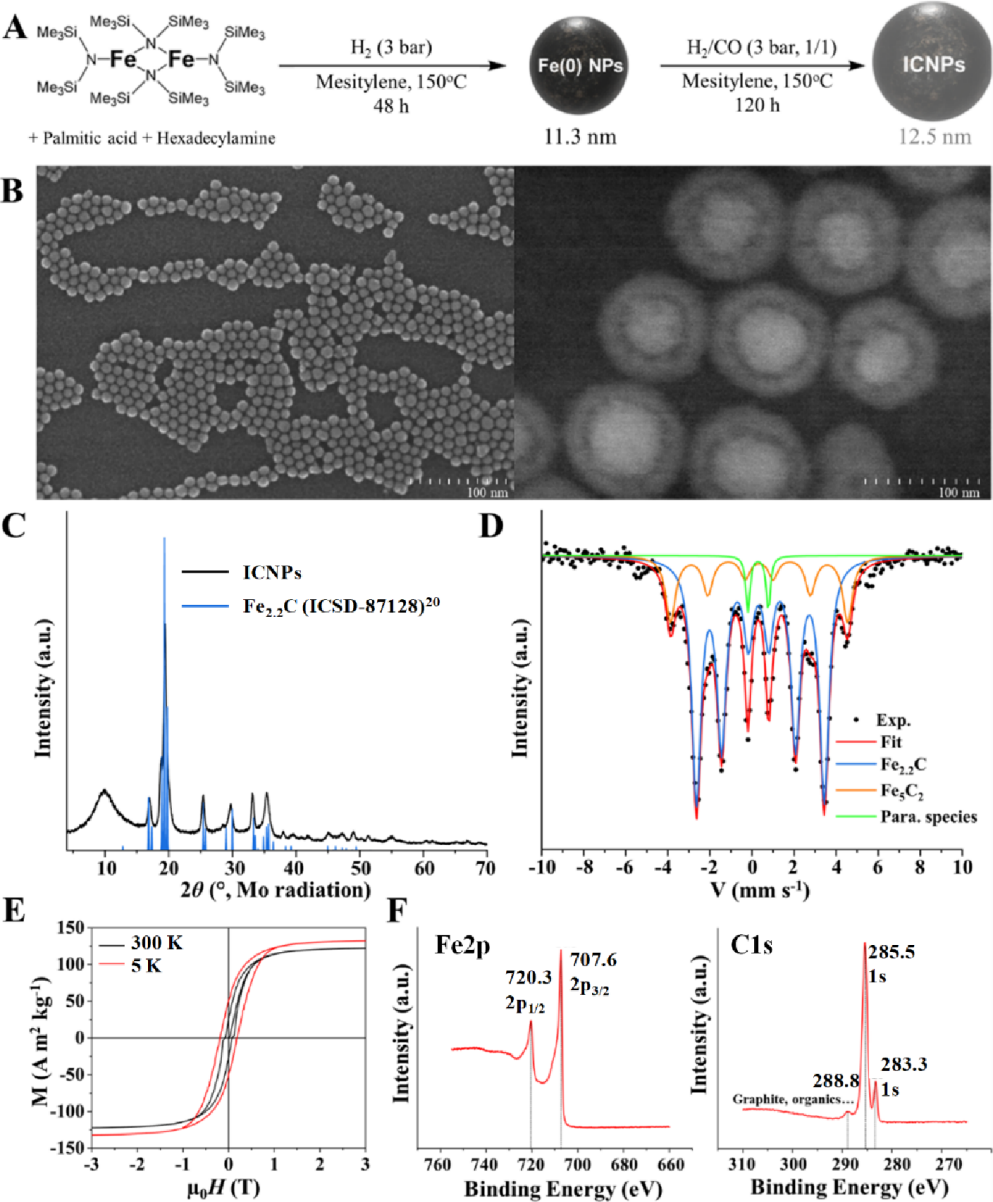
Synthesis and characterization of ICNPs. (A) Organometallic synthetic approach and characterization of ICNPs by (B) scanning electron microscopy (SEM, left) and high angle annular dark field scanning transmission electron microscopy (HAADF-STEM, right), (C) PXRD, with reference to the Fe_2.2_C phase,^20^ (D) ^57^Fe Mössbauer spectroscopy, (E) magnetic measurements at 5 K (red) and 300 K (black), and (F) high resolution XPS of Fe 2p and C 1s.

The powder X-ray diffraction (PXRD) pattern of ICNPs showed the characteristic peaks of the pseudohexagonal Fe_2.2_C carbide phase (Figure 2C). ^57^Fe Mössbauer spectroscopy at 4 K (Figure 2D) confirmed the coexistence of two different iron carbide phases (Fe_2.2_C and Fe_5_C_2_) in expected compositions (76% and 20%, respectively). Vibrating sample magnetometry (VSM) measurements on ICNPs at 300 K demonstrated a more prominent ferromagnetic behavior of these ICNPs at room temperature (Figure 2E) as compared to Fe(0) NPs (Figure S3 and Table S1). These findings are consistent with our previous work.^15^ In addition, the electronic properties of ICNPs were investigated by X-ray photoelectron spectroscopy (XPS) of Fe 2p and C 1s (Figure 2F). The presence of iron as carbide phases was confirmed by Fe 2p_3/2_ and Fe 2p_1/2_ signals at 707.6 and 720.3 eV, respectively. The analysis of the C 1s region also showed a peak at 283.3 eV characteristic of Fe–C bonds besides the typical peak at 285.5 eV of sp^2^ hybridized carbon.^19^

### Catalytic Study

The catalytic performance of ICNPs was investigated for the hydrodeoxygenation of methylbenzoate (**1**) to toluene (**1a**) as a model reaction. The main possible pathways of the hydrodeoxygenation of methylbenzoate are shown in Scheme 1.^21^ The reaction sequence (i) starts by addition of H_2_ and loss of methanol to form benzalydehyde (**E**_**1**_), followed by its hydrogenation to benzylalcohol (**E**_**2**_) and subsequent hydrogenolysis to the desired product **1a**. Path (ii) involves hydrodeoxygenation to benzyl methyl ether (**E**_**3**_) followed by hydrogenolysis to **1a**. As a secondary pathway, the water formed from the main paths could lead to hydrolysis of **1** to benzoic acid as a potential starting point for the analogous sequence **E**_**1**_ – **E**_**2**_ – **1a**. At all stages, generation of ring-hydrogenated compounds must be avoided to achieve high selectivity for the desired aromatic product.

**Scheme 1:**
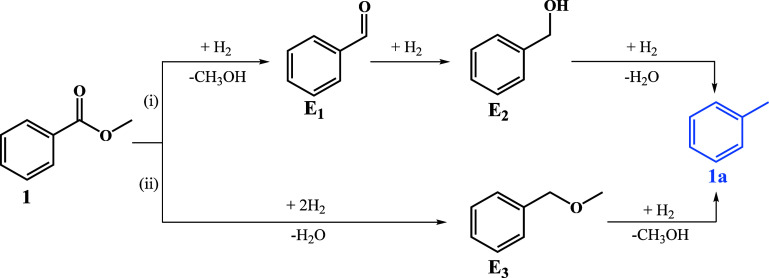
Main Possible Pathways for the Hydrodeoxygenation of Methylbenzoate (**1**) to Toluene (**1a**)

Catalytic experiments were conducted under batch conditions using Fisher-Porter bottles as reactors placed inside a commercial coil for the generation of the ACMF (Figure S4). Methylbenzoate (**1**) was dissolved in a solvent (0.5 mL) and reacted in the presence of the ICNP catalyst (10 mg) under H_2_ (3 bar). The reactions were started by switching on the ACMF generator to a standard set of parameters (*f* = 350 kHz, μ_0_ *H*_max_ = 70 mT). An infrared camera was used to determine the “global temperature” reached by the Fisher-Porter bottle as a result of the dissipation of thermal energy from the ICNP catalyst under the reaction conditions. For reactions conducted under conventional heating, the reaction time monitoring was started once the reaction solution reached the desired temperature. No stirring was implemented, and mixing was ensured only through convection. Products in the liquid and gas-phase were identified and quantified by GC-MS and GC-FID (see details in the Supporting Information).

Solvent selection is crucial for magnetically induced catalysis in liquid phase,^12^ and a series of solvents with different boiling points were tested under standard conditions (Figure 3A). Interestingly, the global temperature reached ca. 200 °C, irrespective of the boiling point of the solvents. The ICNP surface temperature could be estimated to ca. 330 °C by monitoring the boiling of various solvents under these conditions, demonstrating that ICNPs form hot spots in a colder environment (see Table S2 for details). For solvents with relatively low boiling points (heptane and mesitylene), the conversion of **1** remained negligible, presumably due to mass transfer limitation at the NP surface related to the Leidenfrost effect.^18,22^ For higher boiling alkane solvents, substrate conversions of 49–64% were observed after 2 h of reaction, selectively giving toluene (**1a**) as the only product detected. Propylene carbonate and sulfolane were found to be not suitable due to their instability under these conditions (Figure 3A). Poor conversion was observed in tetraethylene glycol, most likely due to the solvent’s strong interaction with ICNPs,^23^ limiting the substrate and H_2_ diffusion to the catalyst surface (Figure 3A).

**Figure 3:**
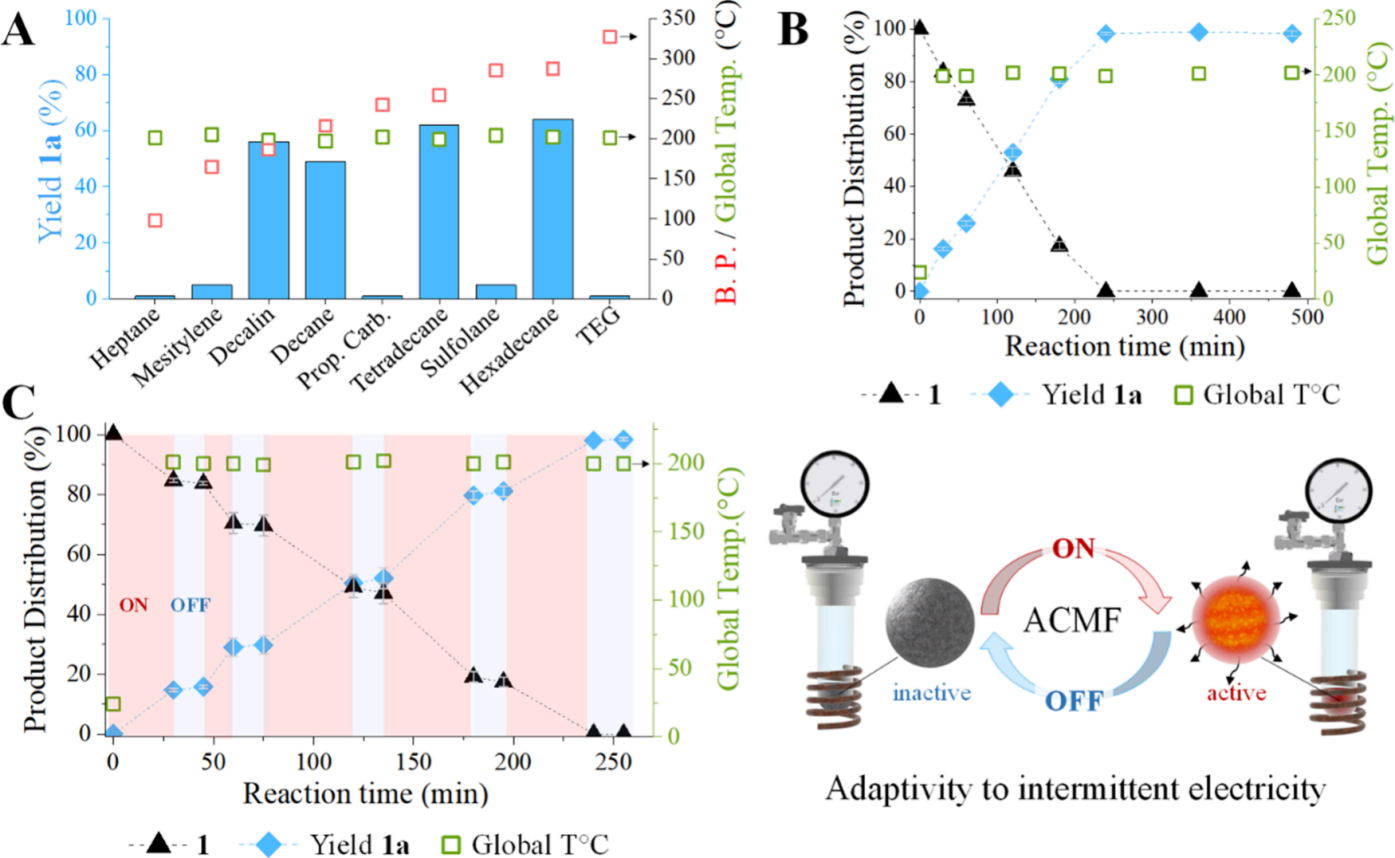
Magnetically induced catalytic hydrodeoxygenation of methyl benzoate (**1**) with ICNPs. (A) Solvent screening for 2 h reactions, (B) time profile in decalin, (C) time profile in decalin recorded while regularly switching ON (red) and OFF (blue) the ACMF power supply. Reaction conditions: methyl benzoate **1** (44.9 mg, 0.33 mmol), ICNPs (10 mg, 0.125 mmol of Fe), solvent (0.5 mL), H_2_ (3 bar), and magnetic field (μ_0_ *H*_max_ = 70 mT, 350 kHz). Product yields determined by GC-FID using tetradecane as the internal standard. Product selectivity is >99% in all cases. Data points are average values of three experiments, and error bars represent standard deviations. Global temperatures (represented by green open squares) were determined by an IR camera.

Recording of a time profile under standard conditions in decalin as solvent showed the progressive conversion of **1** to **1a** with time (estimated initial rate *r*_0_(**1** → **1a**) = 3.4 mmol L^–1^ min^–1^) without detection of any intermediates (Figure 3B and Figures S5 and S6). Methane, CO, and CO_2_ arising from the secondary reactions of methanol under these conditions were detected in the gas phase (Figure S7). Quantitative yield of **1a** was reached after 4 h, and prolonging the reaction time to 8 h did not change the product distribution, outlining the excellent selectivity of the ICNP catalysts toward toluene without any hydrogenation of the aromatic ring. Decreasing the ACMF amplitude to 66, 56, and 50 mT led to milder heating of the ICNPs and slower reactions, as evidenced by incomplete conversions of **1** after 4 h of reaction (75%, 45%, and 30%, respectively, Table S3). Increasing it to 74 mT provided a faster quantitative yield of **1a** without any detectable loss of selectivity. In line with only **1** and **1a** being observed under turnover conditions, the potential intermediates **E**_**1**_, **E**_**2**_, and **E**_**3**_ were converted to toluene at faster apparent initial rates than substrate **1** under identical conditions (*r*_0_(**E**_**1**_ → **1a**) = 6.2 mmol L^–1^ min^–1^, *r*_0_(**E**_**2**_ → **1a**) = 28.2 mmol L^–1^ min^–1^, and *r*_0_(**E**_**3**_ → **1a**) = 8.1 mmol L^–1^ min^–1^), and **E**_**2**_ was not observed as an intermediate in the hydrodeoxygenation of **E**_**1**_ (Figure S6). No conversion of benzoic acid was observed under these conditions, indicating that hydrogenolysis of **1** does not occur to any significant level during the reaction (Table S4).

Importantly, the ICNPs reacted in real time on regularly stopping and restarting the ACMF, resulting in a perfectly concomitant stop and restart of the catalytic activity (Figure 3C). In contrast, the global temperature did not show any variation on the time scale of the switching process, demonstrating that the activation energy for the catalytic reaction is provided solely by the high surface temperature. The extremely fast heating and cooling of the catalyst controlled by magnetic induction demonstrates the system’s adaptivity to fluctuations in electricity supply, a feature of strategic interest when considering the use of renewable energy sources to drive chemical reactions.^12,24,25^ Notably, the catalyst behavior complies with the recently formulated *R* ^3^ rule (reversibility, rapidity, robustness) for adaptivity in catalysis.^24^

Using Fe(0) or Fe_3_O_4_ NPs (see the SI for preparation and Figures S1–S3 and S8 for characterization) of similar size as catalysts in reference experiments (Figure 4A, Figure S7) led to milder global temperatures and poorer conversion (160 °C/53% and 91 °C/0%, respectively), reflecting the expected lower magnetic heating capabilities of these NPs. All three types of NPs were tested under conventional heating to gather insight into the intrinsic activity of different Fe phases for this transformation. The catalysts showed low activity at 200 °C, i.e., the global temperature observed with the ICNPs under magnetic induction. Interestingly, the implementation of stirring did not improve the performance of ICNPs at 200 °C (6% without stirring, 5% with stirring at 700 rpm; Table S5), indicating that mass transfer is not limiting under these conditions. Raising the temperature to 350 °C—20 °C above the estimated surface temperature of ICNPs during magnetically induced catalysis—demonstrated an intrinsic higher activity for the ICNPs (76% yield of **1a**) than for Fe(0) NPs (21% yield of **1a**) and Fe_3_O_4_ NPs (4% yield of **1a**). While the poor activity of iron oxide nanoparticles in hydrodeoxygenation reactions is expected, the intrinsic superior activity of the ICNPs (and in particular of the Fe_5_C_2_ carbide phase located in the NP shell, cf., synthesis and characterization section) as compared to metallic Fe is particularly interesting and echoes with behaviors typically observed in Fischer–Tropsch syntheses^26^ and very recently with ε-Fe_2_C NPs used in reductive amination reaction.^27^ The excellent performance of ICNPs in the selective hydrodeoxygenation of esters contrasts also with the general poor activity and stability of Fe-based heterogeneous catalysts in liquid phase hydrogenation reactions, typically requiring combination with more active metals (e.g., Ni, Cu, Ru, Pd, etc.).^28–31^

**Figure 4:**
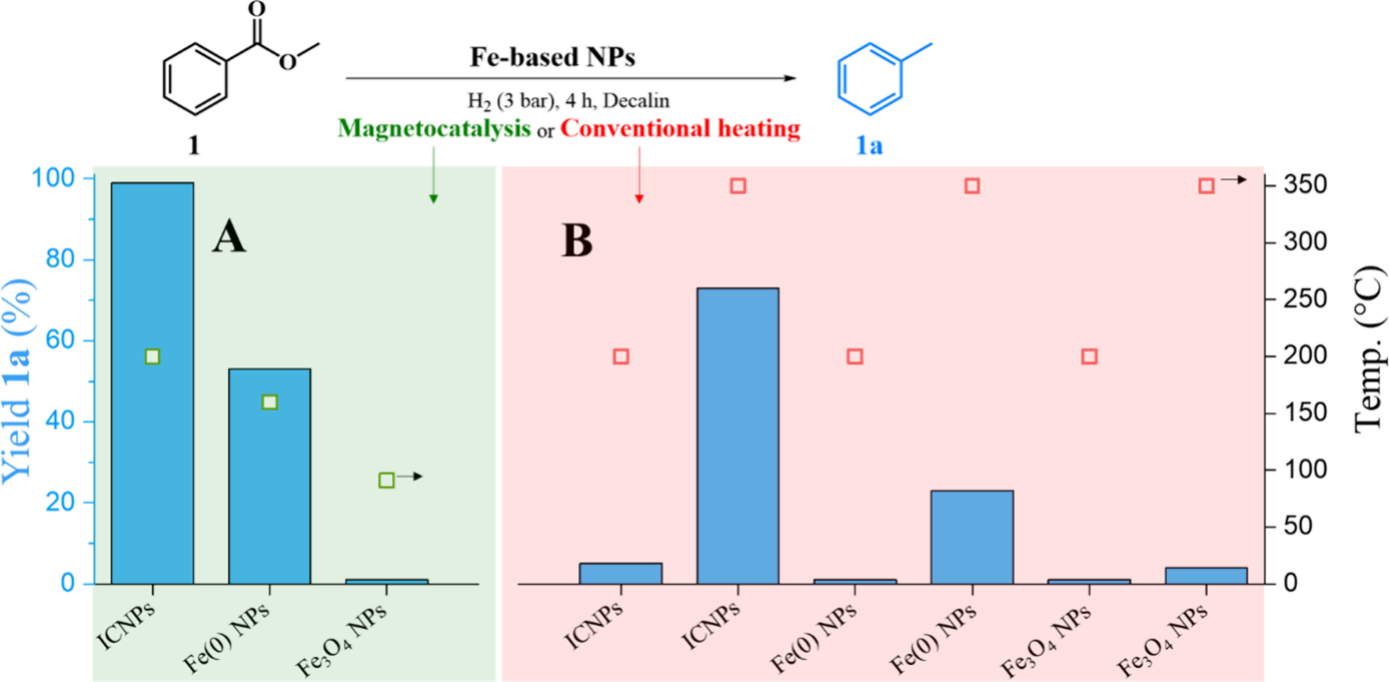
Hydrodeoxygenation of methyl benzoate (**1**) with different Fe-based NPs. (A) Magnetically induced catalysis (350 kHz, 70 mT); (B) conventional heating at 200 and 350 °C. Reaction conditions: methyl benzoate **1** (44.9 mg, 0.33 mmol), Fe-based NPs (10 mg, 0.125 mmol of Fe), decalin (0.5 mL), H_2_ (3 bar), ACMF or conventional heating, 4 h. Product yields were determined by GC-FID using tetradecane as the internal standard. Product selectivity is >99% in all cases. Global temperatures for magnetically induced catalysis were determined by an IR camera.

The energy input required for the magnetocatalytic process using the ICNP catalyst under the standard conditions was determined and compared to the energy input required for conventional heating at 350 °C (see the section “Energy Consumption Analysis” of the SI). Magnetically activated ICNPs consumed 0.175 MJ energy to deliver 19% yield of **1a** in 30 min, while for the same reaction time, conventional heating consumed 1.55 MJ to give comparable catalytic performance (24% yield of **1a**). In addition, 80 min of heating was necessary for the autoclave to reach 350 °C, while magnetically induced activation was almost instantaneous. As a result, the energy efficiency toward product formation is ca. five times higher with magnetocatalysis than with conventional heating at 350 °C even under these nonoptimized laboratory conditions.

The reusability and stability of ICNPs in magnetocatalysis and under conventional heating at 350 °C were investigated adapting the conditions to ensure incomplete substrate conversion in both cases (Figure 5). Owing to their magnetic properties, ICNPs can be easily separated from the product solution after each cycle upon application of an external permanent magnet. The reaction mixture can be simply decanted while the NPs reside in the reactor (see also Figure 7). Satisfyingly, ICNPs could be recycled at least five times under magnetocatalytic conditions without any sign of deactivation (Figure 5A). Characterization of the ICNPs after five cycles by electron microscopy (Figure 5B), PXRD (Figure 5C), ^57^Fe Mössbauer spectroscopy (Figure S9), XPS (Figure S10), and VSM (Figure S11) showed no noticeable change in NP size, dispersion, structural, electronic, or magnetic properties as compared to the pristine ICNPs (see detailed comparison in Table S6). ICP-MS showed negligible leaching of Fe in product solutions (1.5–3.9 μg/L, Table S7). The slight increase in yield throughout the cycles is attributed to the gradual removal of ligands from the ICNP surface, facilitating the access of substrate molecules to the catalytically active sites. This hypothesis is supported by ICP-MS analysis of fresh and used ICNPs that reveals a substantial increase in Fe content from 83.2 wt % to 88.5 wt % after the third reaction cycle (Table S8).

**Figure 5:**
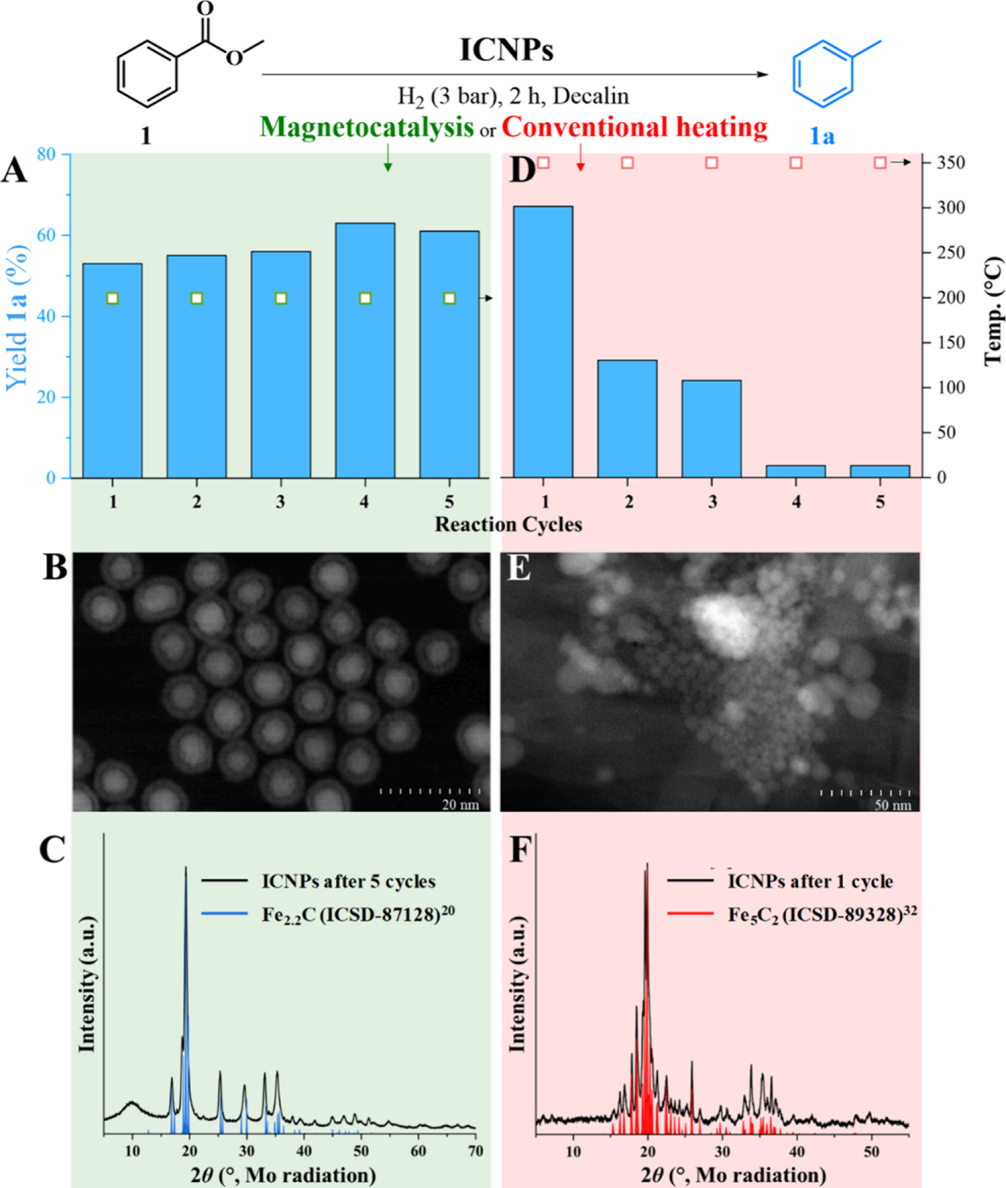
Investigation of the reusability and stability of ICNPs for the hydrodeoxygenation of methyl benzoate (**1**) using magnetically induced catalysis (350 kHz, 70 mT) and conventional heating at 350 °C. (A) Recycling experiment under magnetocatalytic conditions and characterization of ICNPs after 5 cycles by (B) STEM-HAADF and (C) PXRD (20); (D) recycling experiment under conventional heating at 350 °C and characterization of ICNPs after 5 cycles by (E) STEM-HAADF and (F) PXRD.^32^ Reaction conditions: methyl benzoate **1** (44.9 mg, 0.33 mmol), ICNPs (10 mg, 0.125 mmol of Fe), decalin (0.5 mL), H_2_ (3 bar), ACMF or conventional heating, 2 h. Product yields determined by GC-FID using tetradecane as the internal standard. Product selectivity is >99% in all cases. Global temperatures for magnetically induced catalysis were determined by an IR camera.

In contrast, the catalytic performance of ICNPs rapidly declined under conventional heating at 350 °C with a two-third reduction of toluene yield already after the first catalytic cycle (Figure 5D). HAADF-STEM showed aggregated and coalesced ICNPs (Figure 5E), while PXRD revealed a complete transition from the initial Fe_2.2_C crystallographic phase to crystalline Fe_5_C_2_. This resulted in a weaker anisotropy of the used NPs as shown by VSM characterization (Figure S12). Fe leaching was also more substantial than that under magnetocatalytic conditions (Table S9). These results evidence a strikingly superior stability of the catalytically beneficial core–shell structure of the ICNPs used under ACMFs compared to conventional heating at a similar temperature.

Having assessed the efficacy of the approach for the model substrate, the versality of ICNPs as catalysts for the magnetically induced selective hydrodeoxygenation of aromatic esters was explored for a range of possible applications including biomass-derived substrates (e.g., **7**, **8**, **12**, **19**) and polyester model compounds (**18** and **19**) (Figure 6). Substrates **1**–**8** with electron donating substituents on the phenyl were all effectively hydrodeoxygenated, giving the desired tolyl derivatives in excellent yields (81% to 99%). Many of these products find application in synthetic chemistry; for example, **2a** is a building block for the synthesis of indacaterol, a β_2_-adrenoceptor agonist,^33^ and **3a** enters in the synthesis of thiocyanates for biorelevant sulfur-containing scaffolds.^34^ Additional methyl substituents on the ring did not affect the catalytic performance irrespective of their position, as shown from *o*-, *m*-, and *p*-methyl­(methylbenzoate) (**2**–**4**). Interestingly, the hydroxyl group in **7** was substantially cleaved (64% yield of **1a**), which is a promising observation for the potential application of ICNPs to the selective hydrodeoxygenation of phenol derivatives. In contrast, the methoxy functionality was preserved in substrate **8**. Amine substituents were fully tolerated, allowing access to product **12a** (*p*-aminotoluene, 85% yield), which is a synthon for the preparation of pharmaceutically relevant amidines.^35^

**Figure 6:**
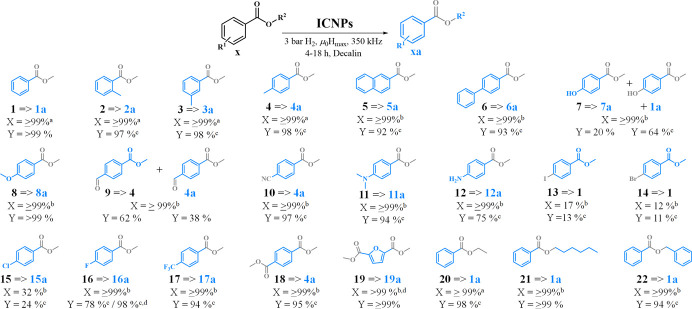
Magnetically induced hydrogenation of various benzylic esters using ICNPs. Reaction conditions: substrate (0.33 mmol), ICNPs (10.0 mg, 0.125 mmol Fe), decalin (0.5 mL), and H_2_ (3 bar). GC product yields determined by GC-FID using tetradecane as the internal standard. X = conversion, Y = yield. ^a^4 h. ^b^18 h. ^c^Y ≠ X, see Schemes S2–S19 for full product distributions. ^d^66 mT.

Most substrates bearing electron-withdrawing functionalities at the phenyl were also hydrodeoxygenated in high yields and selectivities, with the exception of chloro-, bromo-, and iodomethylbenzoate (**13**–**15**), for which hydrodehalogenation was expected and observed. Interestingly, hydrodefluorination could be prevented in substrate **16** by lowering the ACMF amplitude to 66 mT, giving *p*-fluorotoluene (**16a**) in 98% yield as an important intermediate in synthetic chemistry, e.g., for N-arylation^36^ and synthesis of diarylmethanes.^37^ Formyl and nitrile substituents were converted to methyl substituents under these conditions (cf., substrates **9** and **10**, respectively). Importantly, the PET model substrate dimethyl terephthalate (**18**) was converted to *p*-xylene (**4a**, 94% GC yield, 89% isolated yield), substantiating the potential of this catalyst and approach for the conversion of waste PET into valuable aromatic compounds. Similarly, biomass-derived furan-2,5-dicarboxylate (FDCA, **19**) as a model for biobased polyesters was quantitatively converted to dimethylfuran (**19a**, >99%). Replacing the methyl side chain by bulkier substituents (e.g., ethyl (**20**), hexyl (**21**), and benzyl (**22**) Figure S13) still resulted in quantitative yields of the desired products. Notably, a solvent-free 500 mg scale reaction with substrate **22** gave toluene in 90% isolated yield after simply removing the ICNPs with a magnet (Scheme S1, Figure S14). In this case, the substrate to catalyst ratio calculated by considering the total amount of Fe and the estimated amount of Fe atoms available at the surface of the ICNPs (see the SI for details) reached 21 and 350, respectively.

Encouraged by the promising performance of magnetically activated ICNPs in the hydrodeoxygenation of polyester model substrates **18** and **19**, the chemical recycling of PET pieces cut from a commercial coffee cup was attempted (Figure 7). Satisfyingly, 70 wt % of the PET was converted, giving selectively *p*-xylene, while the corresponding ethylene glycol part of the PET structure was converted mainly into ethane and methane detected in the gas phase (Figure S15). The aromatic *p*-xylene can be reintegrated into the chemical value chain to generate terephthalic acid as PET monomer (closed-loop recycling) or for other uses, e.g., as additive to fuels (open-loop recycling).^38^ The gaseous light alkanes can be used directly as energy molecules or fed back into refinery processes, e.g., into an ethylene cracker for regenerating ethylene glycol. Notably, the ICNP catalyst could be easily separated at the end of the reaction from the product mixture due to its ferromagnetic properties.

**Figure 7:**
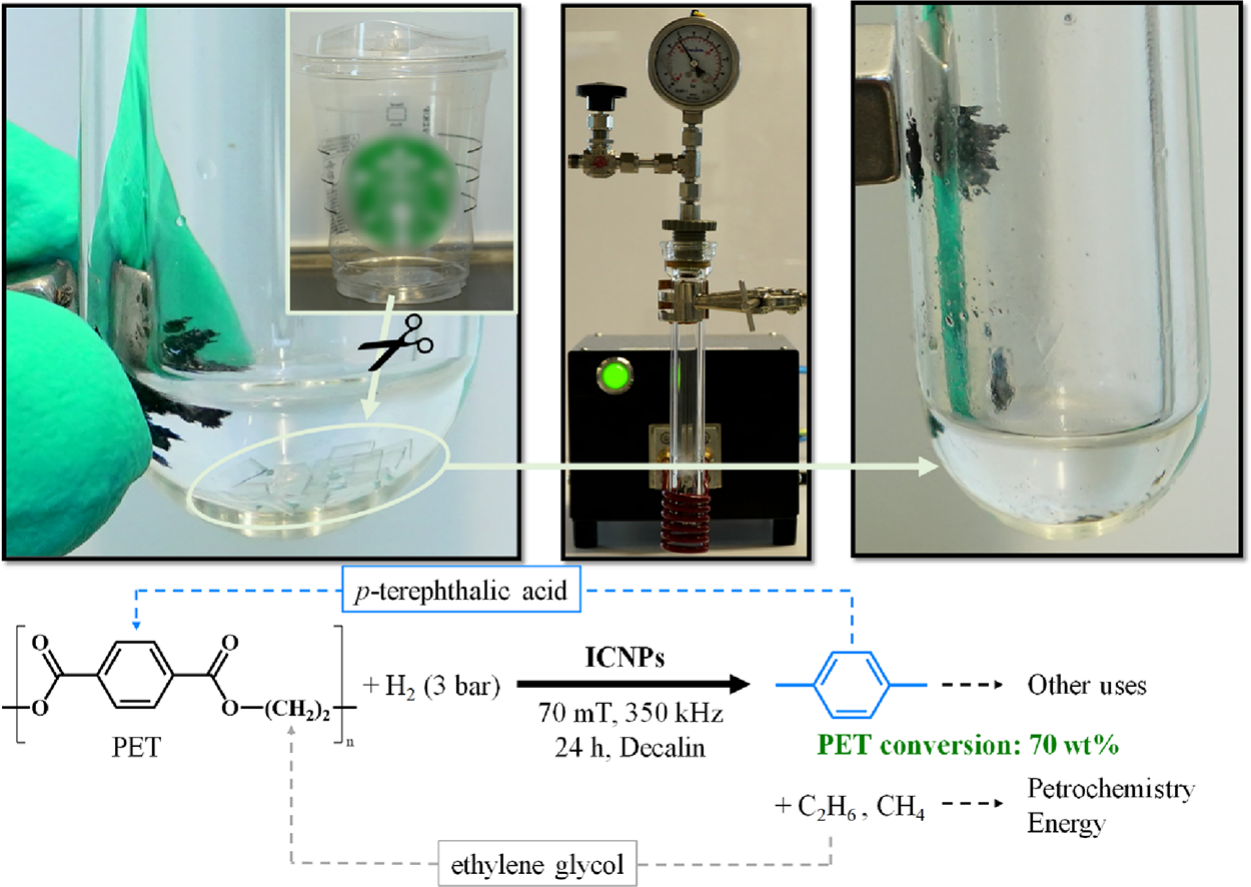
PET to chemicals using magnetically induced catalysis and ICNPs. PET samples originate from a real commercial plastic cup cut into small pieces. At the end of the reaction, ICNPs are easily separable from a magnet. Reaction conditions: PET (50 mg), ICNPs (20 mg), 3 bar H_2_, 70 mT, 350 kHz, 24 h, decalin (0.5 mL).

## Conclusions

Ferromagnetic monodomain iron carbide nanoparticles (ICNPs) are activated by magnetic induction by using an alternating current magnetic field (ACMF) to catalyze hydrodeoxygenation of benzylic esters in a highly active and selective manner. The innovative approach of magnetocatalysis enables valuable synthetic pathways to methyl-substituted aromatic compounds under mild H_2_ pressure (3 bar) and moderate reactor temperature (ca. 200 °C) and opens new possibilities for the chemical recycling of polyester materials. It also allows the introduction and efficient use of green electricity in catalytic processes while coping with the intermittency of renewable electricity. Potential advantages over conventional thermal catalytic approaches demonstrated in this work include (i) lower global temperatures, (ii) higher energy efficiency, (iii) adaptability to energy fluctuations, (iv) easy catalyst separation, and (v) improved catalyst stability.

This work may pave the way toward practical ester and polyester hydrodeoxygenation with magnetically activated earth-abundant Fe-based catalysts using renewable H_2_ at the laboratory and production scales. In addition, the observed chemical and process benefits are of significant general interest, further encouraging the exploration of the emerging field of magnetically induced catalysis in research and industry.

## Supplementary Material



## Data Availability

Methods, supplementary tables and figures are provided in the Supporting Information. Source data are provided on the Edmond repository of the Max Planck Society and available at https://doi.org/10.17617/3.UWVM25.

## Notes

Materials and Methods section, estimation of surface Fe atoms, energy consumption analysis, supplementary figures and tables including catalyst characterization data (electron microscopy, PXRD, VSM, Mössbauer, XPS) and catalytic data ( magnetocatalytic setup, GC analysis, time profiles, catalytic performance data, variations of reactions conditions, elemental analysis, isolated yields, NMR spectra, and GC for PET recycling (PDF)
