# Supplementary material for: Magnetically Induced Iron-Catalyzed Hydrodeoxygenation of Benzylic Esters and Polyesters
Source: J Am Chem Soc. 2025 Sep 11;147(38):34758–66. doi: 10.1021/jacs.5c10464 (PMC12464968; doi:10.1021/jacs.5c10464)
Supplement: Supplementary file 1 [file ja5c10464_si_001.pdf]

Supplementary Materials for

Magnetically Induced Iron-Catalyzed Hydrodeoxygenation of Benzylic Esters and Polyesters

Sihana Ahmedi[1,2], Lise-Marie Lacroix[3,4], Derya Demirbas,[5] Daniel J. SantaLucia,[1] Claudia Weidenthaler[5], Walid Hetaba[1], Walter Leitner[1,2]*, Alexis Bordet[1]*

Corresponding author: walter.leitner@cec.mpg.de; alexis.bordet@cec.mpg.de

[1]Max Planck Institute for Chemical Energy Conversion, Mülheim an der Ruhr, Germany

[2]Institute of Technical and Macromolecular Chemistry RWTH Aachen University, Aachen, Germany

[3]Université de Toulouse, Laboratoire de Physique et Chimie des Nano-Objets, UMR 5215 INSA, CNRS, UPS, 135 avenue de Rangueil 31077 Toulouse, France

[4]Institut Universitaire de France (IUF), 103 boulevard Saint Michel, 75005 Paris, France

[5]Max-Planck-Institut für Kohlenforschung, Mülheim an der Ruhr 45470, Germany

The PDF file includes:

Materials and Methods 2

Experimental 2

Energy Consumption Analysis 6

Supplementary Figures and Tables 7

Isolated Yields 22

References 25

Materials and Methods

Materials

All syntheses were performed under argon either by using Schlenk techniques or in an argon-filled glove box. ICNPs and Fe(0) nanoparticles are sensitive to oxidation by air, and were thus handled under argon (using Schlenk techniques or a glovebox) at all time. Solvents were purified through a solvent purification system (MBraun-SPS-7) or dried over activated 4 Å molecular sieves, then degassed and preserved under an argon atmosphere before use. Hexadecylamine (HDA, 99%), palmitic acid (PA, 99%) and $FeCl_2$ anhydrous (98%) were purchased from Sigma-Aldrich and abcr GmbH. CO and H_2 gas were purchased from Air Liquide. Esters for the substrate scope were purchased from the local suppliers (e.g. Sigma-Aldrich, abcr, Alfa Aesar) and used without further purification.

Experimental

Characterization Techniques

X-ray photoelectron spectroscopy (XPS) samples were prepared in an Argon-filled glovebox. Subsequently, these samples were carefully transferred to a mobile analysis chamber inside the glovebox, which was then connected to the XPS devices. The XPS experiments were recorded using monochromatized Al-Kα (1487 eV) radiation and a Phoibos NAP-150 hemispherical analyzer from SPECS GmbH. High resolution spectra were collected using 20 eV pass energy. The instrument work function was calibrated to give an Au 4f7/2 metallic gold binding energy of 83.96 eV. Instrument base pressure was 5 * 10-8 mbar. TEM images were recorded on a Hitachi HF2000 operating at 200 kV, and SEM was performed using Hitachi microscope (Model S-5500) working at 30 kV. EDX detector is Thermo Scientific UltraDry (SDD) at the shared facility with the Max Planck Institute for Coal research (KoFo). Samples for electron microscopy were prepared by depositing the powder onto a copper grid with an amorphous carbon support film. To determine the NPs size, the particles were measured using ImageJ with a count of at least 200 NPs. The Gaussian fit of the frequency of size ranges gave the size distribution. For ICP-MS measurements, the samples were digested with 8 mL HNO_3 in the CEM microwave MARS 6. The microwave program is as follows: Power: 1500 W, ramp up to 200 degrees for 30 min, hold time 15 min, temperature 200 ºC. The samples were then measured using the Shimadzu ICPMS-2030 ICP-MS device. Magnetic characterization. Magnetic measurements were performed using a Quantum Device PPMS Evercool II with the vibrating sample magnetometer (VSM) module. About 10 mg of dry powder was introduced in a polypropylene holder under inert atmosphere. The magnetization versus magnetic field measurements (hysteresis loop) were conducted at 300 with an external magnetic field of ± 3T and at 5K after cooling the sample under an external magnetic field of +3T. This procedure should allow to see the presence of a surface oxidation through the appearance of an exchange bias at low temperature. None of the sample exhibited such a shift in this study. The absolute magnetization measured in emu (cgs unit) were normalized by the sample weight (g unit) to retrieve values in $Am^2.kg^{-1}$ ^{57}Fe Mössbauer measurements were carried out in-house. In the glovebox, an iron sample was loaded into a teflon sample holder (ca. 1 cm x 1 cm), and if needed, mixed with eicosane or boron nitride to fill any additional space. The ^{57}Fe Mössbauer spectra were collected on a spectrometer with conventional alternating constant acceleration of the γ-ray source. The sample temperature was controlled using an Oxford Instrument Variox for zero field measurements or a Cryogen-Free Magnet (CFM) with integrated variable temperature insert (VTI) perpendicular to the γ beam. The ^{57}Co source on Rh matrix (1.8 GBq) rests at room temperature in the gap of the magnet system at the zero field

position, by using a reentrant bore. Isomer shifts are quoted relative to α-iron at 300 K. The ^{57}Fe Mössbauer were simulated and fitted with MX program (written by Dr. Eckhard Bill and available by email to: daniel.santalucia@cec.mpg.de). Product analysis was achieved by GC-FID (gas chromatography coupled with flame ionization detection) on a Shimadzu GC 2030 equipped with a CP-WAX-52CB. GC-MS (gas chromatography coupled with mass spectrometry) on a Shimadzu QP 2020 instrument used with its internal compound library for identification and identification of unknown products. Product peak area to the peak are of tetradecane standard were used for product quantification for GC-FID. Solution state NMR spectra were recorded on a BRUKER AscendTM 400/600 at room temperature (^{1}H: 400/600 MHz, ^{13}C: 100 MHz). First order spin multiplicities are abbreviated as s = singlet, d = doublet, t = triplet, q = quartet. Couplings of higher order and overlapping signals are denoted as multiplet (m). The coupling constants (J) are given in Hertz (Hz) and the chemical shifts (δ) are expressed in ppm, relative to TMS at 25 °C. X-ray diffraction data were collected on a Stoe STADI P transmission diffractometer in Debye-Scherrer geometry ($Mok_{\alpha1}$: 0.7093 Å) with a primary monochromator (curved germanium (111)) and Mythen 1K position sensitive detector. The samples were filled in a glove box into glass capillaries (Ø 0.3 mm) and sealed. Data were collected in the range between 2 to 70° 2θ with a step width of 0.015° 2θ.

ACMF generator and reactor

A commercial ACMF generator (UPT-n5) from the company UltraFlex Power Technologies was used for all experiments involving magnetic induction heating. The generator can operate at a maximum power of 5 kW. The commercial copper coil used (6 turns coil, 25 mm internal diameter, 42 mm length) was water-cooled by a chiller (TAEevo Tech MINI 03/10) and operated at a frequency of 350 kHz and a field amplitude adjustable in the [0-75 mT] range.
Catalytic experiments were conducted using commercially-available thick-walled Fisher-Porter bottles (22 mm external diameter, 160 mm length). For this study, the FP bottles were bought from the company Avitec-artec.

Synthesis

Synthesis of ICNPs

Iron carbide nanoparticles (ICNPs) were prepared following a previously reported procedure (*15*). $\{Fe[N(SiMe_3)_2]_2\}_2$ was also prepared according to the reported protocol (*39*). They were obtained in two steps through the carbidization of preformed Fe(0) nanoparticles:

$\{Fe[N(SiMe_3)_2]_2\}_2$ precursor

In an argon-filled glovebox, anhydrous $FeCl_2$ (1.9 g, 15.0 mmol) was dissolved in dried and degassed diethyl ether (60 mL). The suspension was cooled to 0 °C in an ice bath, which was shortly followed by subsequent dropwise addition of a solution of $LiN(SiMe_3)_2$ (5.0 g, 30.0 mmol) in diethyl ether (90.0 mL). The solution was then left to warm up to room temperature under stirring overnight. After 18 h, the volatile components were removed under vacuum, which resulted in a concentrated mixture to form a dark green oil. The Schlenk flask was then connected to a distillation setup to which the product was distilled under reduced pressure (below 0.05 mbar and no cooling water was necessary), and the oil bath was set first to 35 °C to eliminate any ether remaining, then to 90-105 °C to start the distillation process. The product was then stored at -30 °C in the glovebox freezer (the green oil solidified to a green solid in 4.5 g, 80% yield).

Fe(0) NPs

In a Fisher-Porter bottle (in the glovebox), the mixture of solutions that were added in order are: palmitic acid (PA) (1.3 eq, 2.6 mmol, 666.4 mg) and hexadecylamine (HDA) (1.0 eq, 2 mmol, 483 mg) were added to a green solution of $\{Fe[N(SiMe_3)_2]_2\}_2$ (1 mmol (2 mmol Fe), 753 mg) in 40 mL of distilled and degassed mesitylene in a Fisher-Porter bottle. The bottle was then pressurized with dihydrogen (3 bar) and placed in an oil bath at 150 °C for 48 h under vigorous magnetic stirring. The nanoparticles were then recovered by decantation, which was assisted by a permanent magnet, followed by washing 3 x 10 mL with toluene and 3 x 10 mL with THF. The resulting nanoparticles were dried under vacuum and stored in the glovebox.

Carbidization of Fe(0) NPs

The Fe(0) nanoparticles (100 mg, 0.45 mmol of Fe) were dispersed in a Fisher-Porter tube containing 9 mL mesitylene that was stored in the glovebox. The bottle was pressurized with CO / H_2 (2 bar / 2 bar), followed by heating and stirring at 150 °C for 120 h. The obtained nanoparticles were recovered by decantation (assisted by permanent magnets), washed with 3 x 5 mL toluene, then dried under vacuum and stored in the glovebox.

Fe_3O_4 NPs

Fe_3O_4 NPs were obtained by exposing the Fe(0) NPs to air at room temperature for 16 h, which resulted in their full oxidation.

<u>Catalysis</u>

<u>Magnetically-induced catalysis (i.e. with ACMFs)</u>

Typically for all magnetocatalytic experiments, ICNPs (10.0 mg, 0.125 mmol Fe), a solvent (0.5 mL), and the substrate (0.33 mmol) were placed in a Fisher-Porter tube. The Fisher-Porter tube was degassed, and pressurized with the desired pressure of hydrogen (3 bar). The reaction mixture was placed at the center of a copper coil at the desired magnetic ACMF amplitude and fixed frequency of 350 kHz. Once the reaction was finished, the reactor was cooled and vented. After filtration, the reaction mixture was analyzed by GC-FID using tetradecane as the internal standard.

<u>Catalysis with conventional heating</u>

For catalytic experiments under conventional heating at 200 °C, the catalyst (0.125 mmol of Fe), decalin (0.5 mL), and substrate (0.33 mmol) were placed in a Fisher-Porter tube. The Fisher-Porter tube was degassed, and pressurized with the desired pressure of hydrogen (3 bar). The Fisher-Porter tube was placed in an oil bath and the reaction was performed at the desired temperature. Once the reaction was finished, the Fisher-Porter tube was cooled and vented. After filtration, the reaction mixture was analyzed by GC-FID using tetradecane as the internal standard.

For reactions performed at higher temperatures (350 °C), stainless steel autoclaves heated in aluminum heating blocks were used.

N.B. The reaction time monitoring started once the reaction solution reached the desired temperature, as determined by a thermocouple.

<u>Kinetic study</u>

Time profiles were collected following similar protocols, by performing individual reactions for each selected time. Initial reaction rates were determined by plotting products concentrations as a

function of time, using data points collected at short reaction times (i.e. low substrate conversion, kinetically-controlled regime), see fig. S8.

Recycling experiments

ICNPs (10.0 mg, 0.125 mmol Fe), decalin (0.5 mL), and methyl benzoate (44.9 mg, 0.33 mmol) were placed in a Fisher-Porter bottle. The Fisher-Porter bottle was degassed, and pressurized with the desired pressure of hydrogen (3 bar). The reaction mixture was placed at the center of a copper coil at the desired ACMF amplitude of 70 mT and fixed frequency of 350 kHz for 2 h. Once the reaction was finished, the reactor was cooled and vented. After filtration, the reaction mixture was analyzed by GC-FID using tetradecane as the internal standard. For the next cycle, fresh portions of the methyl benzoate (44.9 mg, 0.33 mmol) and decalin (0.5 mL) were added and the reaction mixture was performed again. This procedure was repeated for each catalyst cycle by pressurizing the Fisher-Porter bottle with 3 bar of hydrogen.

In similar protocol, for the conventional heating recycling experiments at 350 °C, ICNPs (10.0 mg, 0.125 mmol Fe), decalin (0.5 mL), and methyl benzoate (44.9 mg, 0.33 mmol) were placed in a stainless-steel autoclave. The reaction mixture was placed on aluminum heating blocks at 350 °C for 2 h. Once the reaction was finished, the reactor was cooled and vented. After filtration, the reaction mixture was analyzed by GC-FID using tetradecane as the internal standard. For the next cycle, fresh portions of the methyl benzoate (44.9 mg, 0.33 mmol) and decalin (0.5 mL) were added and the reaction was performed again. This procedure was repeated for each catalyst cycle by pressurizing the Fisher-Porter bottle with 3 bar of hydrogen.

After each cycle, the catalyst was washed 3 times with decalin (1 mL) to prevent the potential accumulation of substrate and products.

Supplementary Text:

Estimation of surface Fe atoms in ICNPs

Assumptions: spherical ICNPs, distribution of Fe atom on the surface symmetric in all directions; mean NPs size = 12.5 nm:

$$r_{ICNPs} = 6.25\, nm$$
$$V_{ICNPs} = \frac{4}{3}\pi(6.25)^3 = 1022.65\, nm^3$$

Shell volume containing first Fe layer given that: $r_{atomic\, Fe} = 0.126\, nm \rightarrow V_{shell} = \frac{4}{3}\pi((6.25)^3 - (6.25 - 0.126)^3) = 60.61\, nm^3$

$$\%surface\ Fe = \frac{V_{shell}}{V_{ICNPs}} x\ 100 = \frac{60.61}{1022.65} x\ 100$$
$$= 5.93\ \%\ of\ Fe\ atoms\ in\ ICNPs\ are\ on\ the\ surface$$

Total particle volume: V_{ICNPs} = 1022.65 nm^3
Surface Fe shell volume: V_{shell} = 60.61 nm^3
%surface Fe atoms = 6%

Energy Consumption Analysis

Energy consumption analysis for the HDO of **1** using ICNPs activated by ACMF (70 mT, 350 kHz) or conventional heating at 350 °C:

With magnetically-induced catalysis (ACMF 70 mT, 350 kHz), the ICNPs absorb ca. 96 J s^{-1} of power input (SAR of ICNPs estimated to ca 8500 W/g_{Fe} under these conditions) (17), and releases it as thermal energy, accounting for ca. 1.4 MJ of energy over 4 h. The catalyst reached almost instantaneously its working temperature (estimated to 350 °C). Under these conditions (70 mT, 350 kHz, 3 bar H_2, 4 h), substrate **1** was fully converted, and product **1a** was obtained in quantitative yield.

In contrast, with conventional heating the autoclave took 80 min to reach the target 350 °C. After operating it for 4 hours at 350 °C, a total energy consumption of 4.3 MJ was measured by a power meter. Under these conditions, the yield of **1a** was 73%. Thus, for lower catalytic performance, conventional heating consumed ca. 4.3 MJ, while the magnetically heated catalyst consumed only 1.4 MJ (see Table below for a summary).

	Magnetic heating (70 mT, 350 kHz)	Conventional heating (350 °C)
Time to target T °C (h)	0	1.2
Reactor T °C	200	350
Reaction time (h)	0.5	0.5
Energy input to reactor (MJ)	0.175	1.55
Yield of **1a** (%)	19	24
Energy efficiency toward product formation (mmol MJ^{-1})	0.243	0.0510

Supplementary Figures and Tables

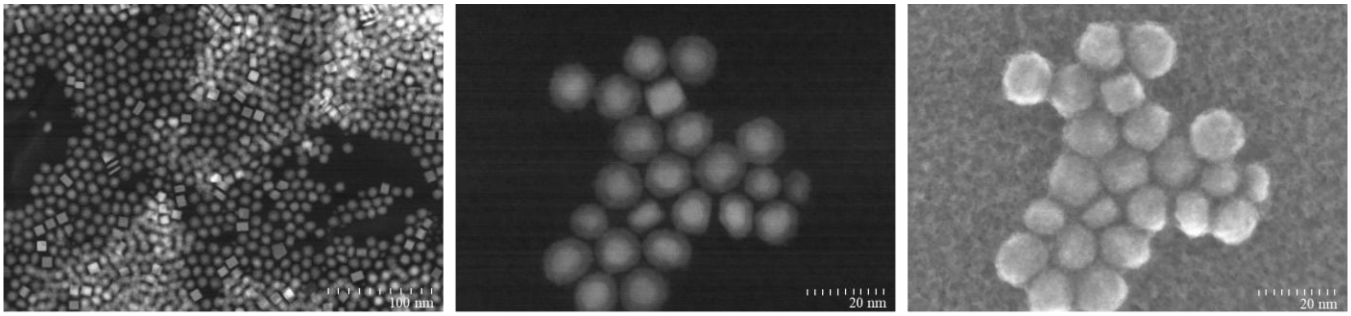

fig. S1. TEM/SEM analysis of Fe(0) nanoparticles (11.3 ± 1.3 nm).

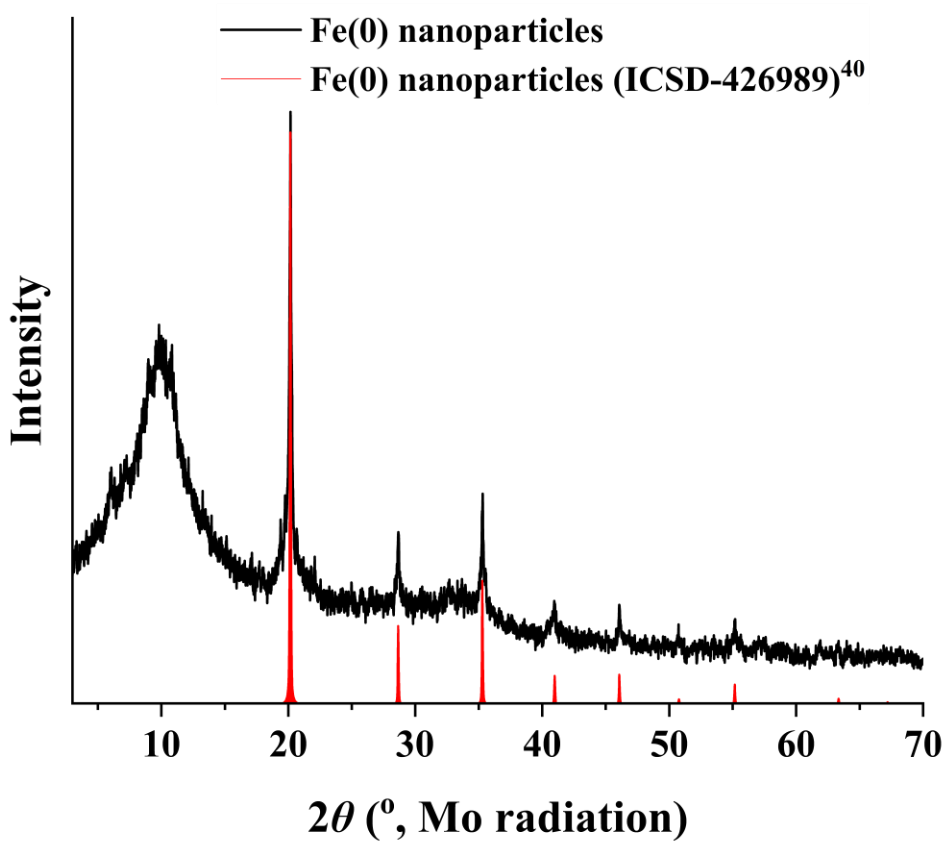

fig. S2. PXRD analysis of Fe(0) nanoparticles, with Fe(0) reference (*40*).

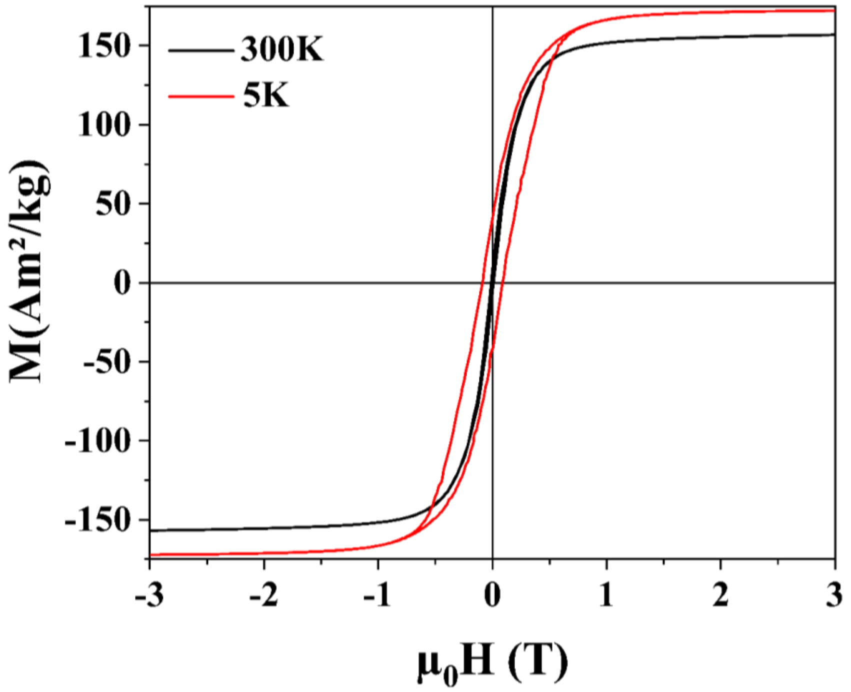

fig. S3. Magnetic characterization of Fe(0) NPs at 300 K (black) and 5 K (red).

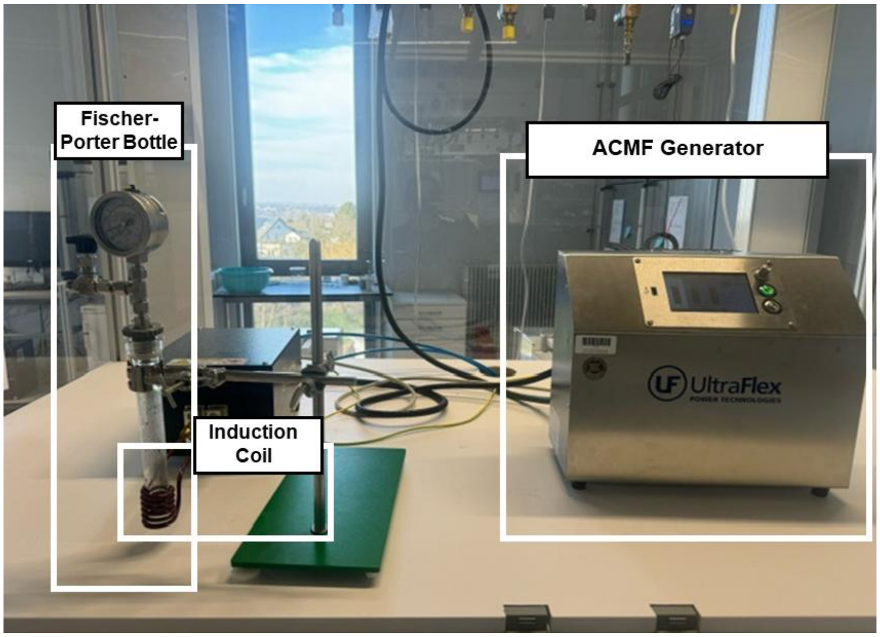

fig. S4. Picture of the magnetocatalytic set up.

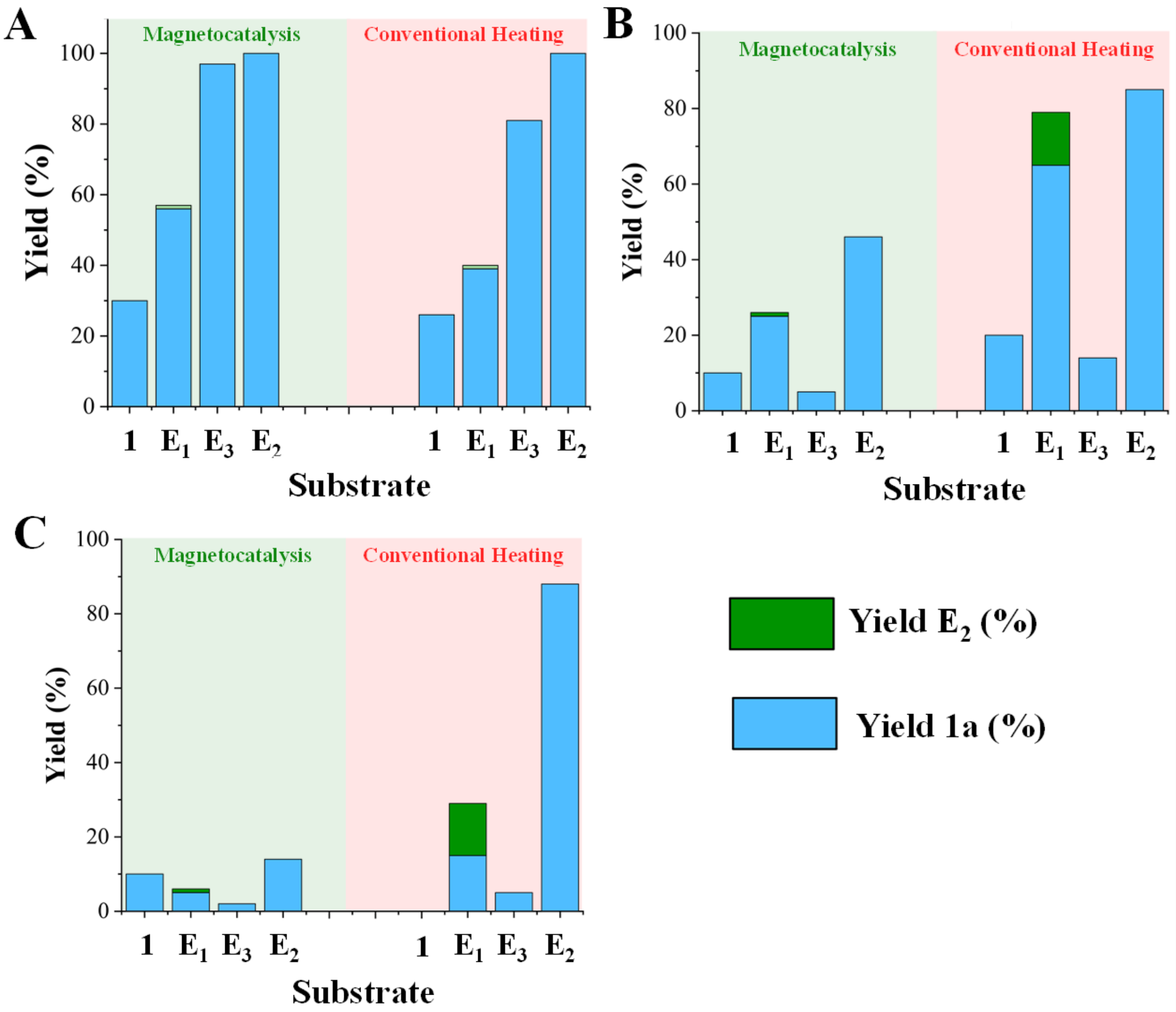

fig. S5. Catalytic performance of Fe-based NPs for different substrates: **1** methyl benzoate, $\mathbf{E_1}$ benzaldehyde, $\mathbf{E_3}$ (methoxymethyl)benzene or $\mathbf{E_2}$ benzyl alcohol (0.33 mmol) using Fe-based nanoparticles catalysts: a) ICNPs, b) Fe(0) NPs, and c) Fe_3O_4 NPs (0.125 mmol of Fe). Reaction conditions for magnetocatalysis: decalin (0.5 mL), H_2 (3 bar), ACMF (70 mT, 350 kHz) in 1 h. Reaction conditions for conventional heating: decalin (0.5 mL), H_2 (3 bar), 350 °C in autoclave in 1 h. Product yields determined by GC-FID using tetradecane as the internal standard.

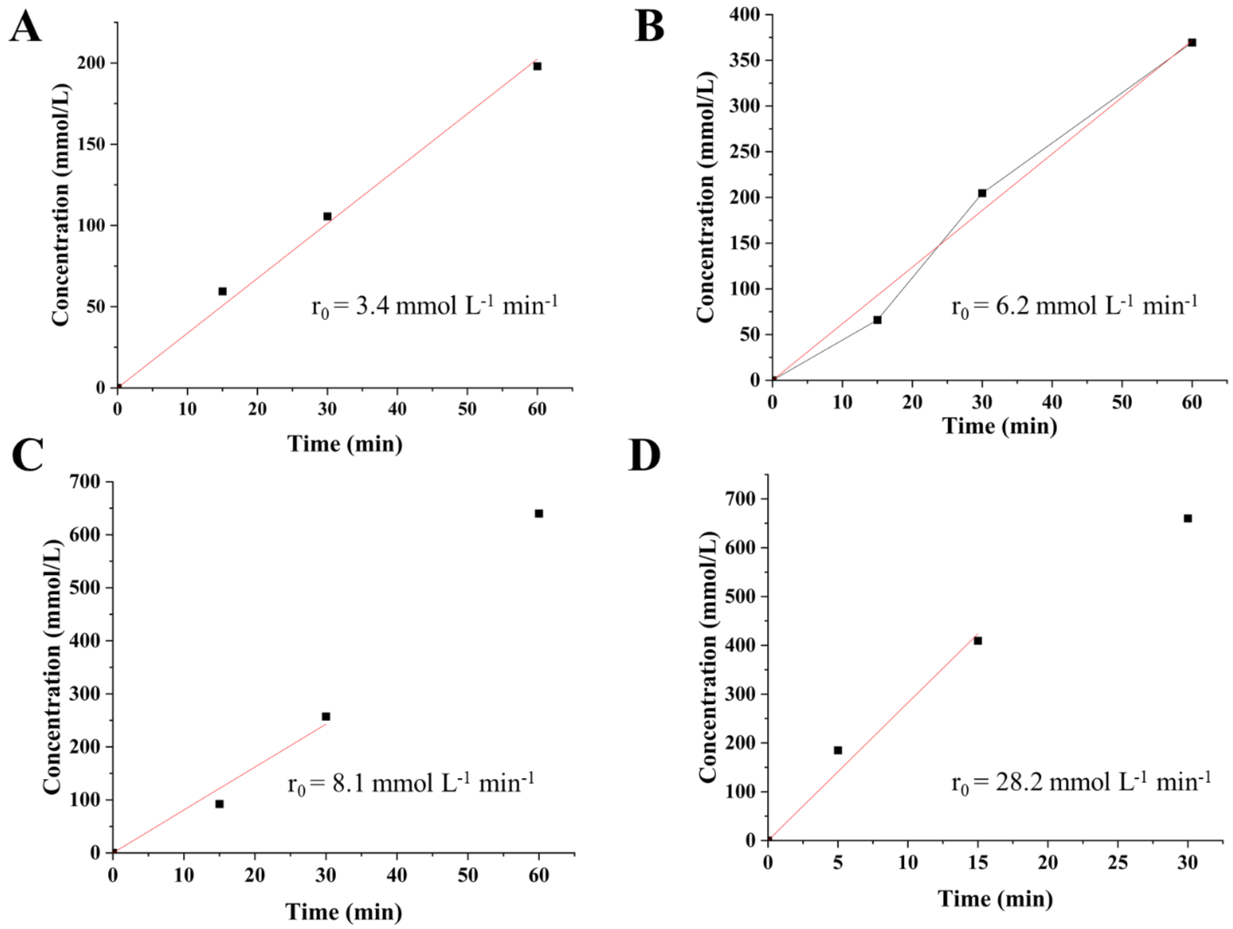

fig. S6. Time profile for the HDO of different substrates into toluene (**1a**) with ICNPs: A) **1** methyl benzoate, B) **E1** benzaldehyde, C) **E3** (methoxymethyl)benzene, D) **E2** benzyl alcohol. Reaction conditions: ICNPs (10 mg, 0.125 mmol of Fe), substrate (0.33 mmol), decalin (0.5 mL), H_2 (3 bar), ACMF (70 mT, 350 kHz) at different times. Product yields determined by GC-FID using tetradecane as the internal standard.

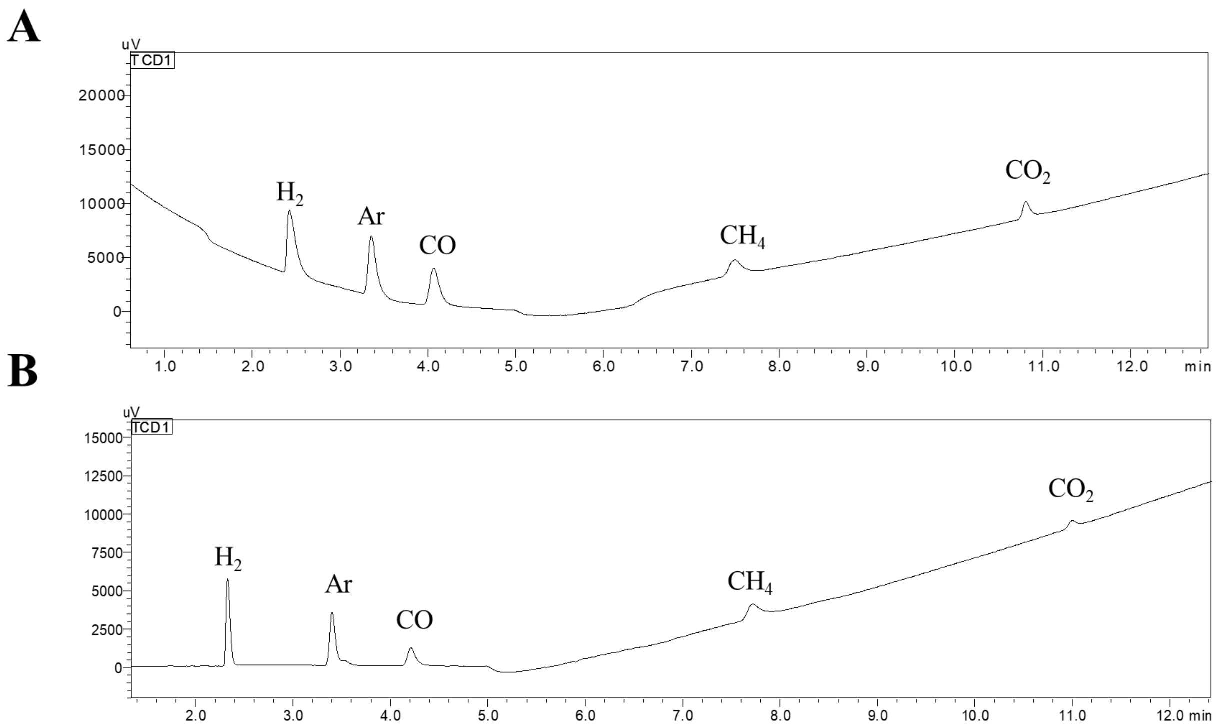

fig. S7. GC-Headspace, gas phase analysis: a) after 4 h reaction with methyl benzoate (0.33 mmol) under standard conditions, b) after 1 h reaction with MeOH (0.33 mmol) under standard conditions.

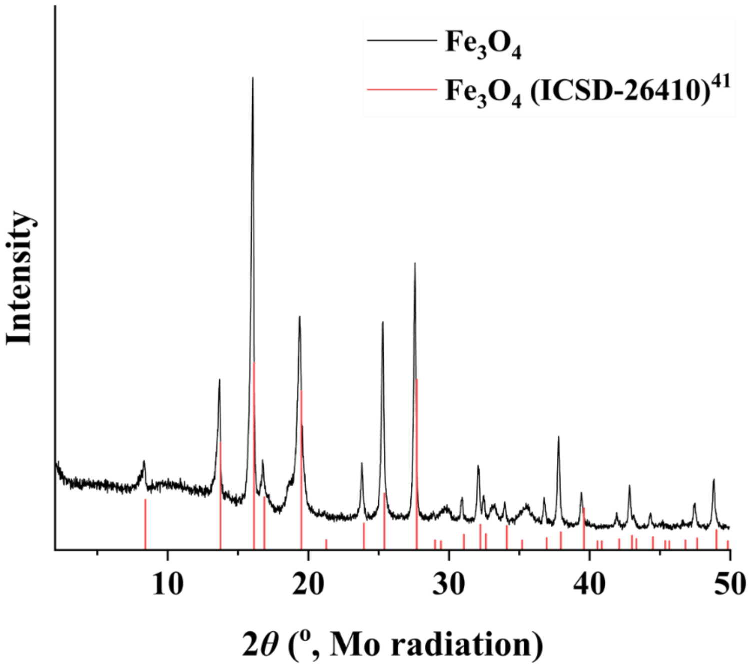

fig. S8. Powder X-ray diffraction of Fe(0) NPs after 16 h of air exposure at room temperature, showing the formation of Fe_3O_4 NPs. (with Fe_3O_4 reference (*41*)).

A

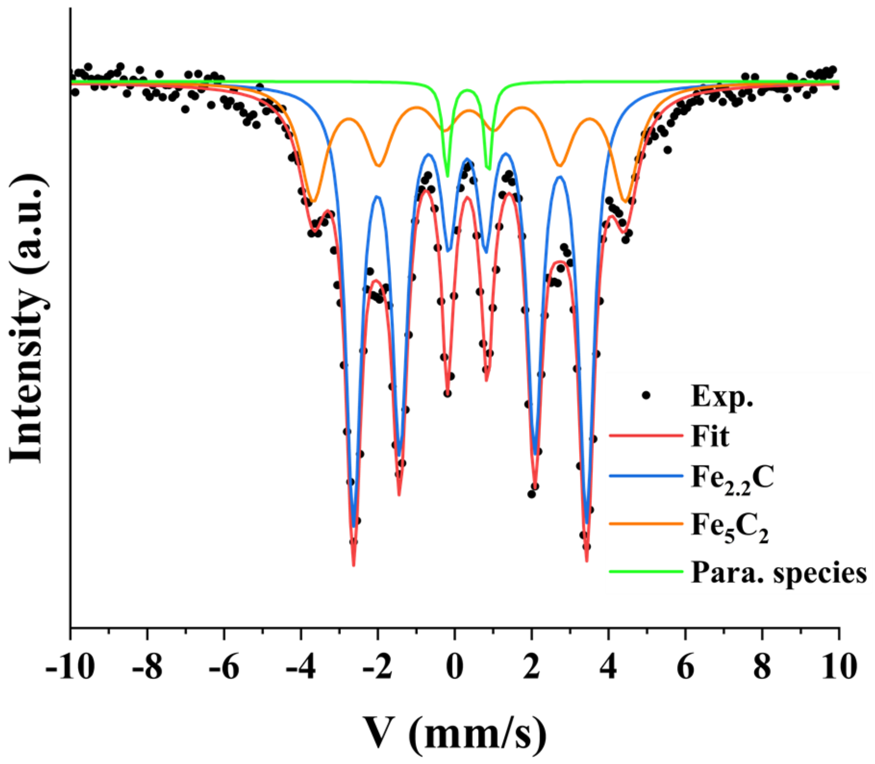

B

Phases	δ (mm/s)	Q (mm/s)	μ_0Hmax (T)	Quantity (%)
Paramagnetic species	0.5	0	0	3
$Fe_{2.2}C$	0.35	0	18.826	68
Fe_5C_2	0.37	0	25.222	28

fig. S9. Low temperature (4 K) Mössbauer analysis of ICNPs after 5 cycles of magnetocatalytic hydrodeoxygenation of methyl benzoate (**1**) to toluene (**1a**). (a) ^{57}Fe Mössbauer spectrum, (b) fitting parameters and the resulting nanoparticles composition.

As shown in fig. S6, quantifying the paramagnetic species compared to both nanoparticle phases is potentially a little bit misleading, due to the very different Mössbauer temperatures for these types of species. A very small amount of Fe(0) based on the peaks present at +/- 5.5 mm/s that wasn't modeled due to the inability to resolve it from the rest of the overlapping signals.

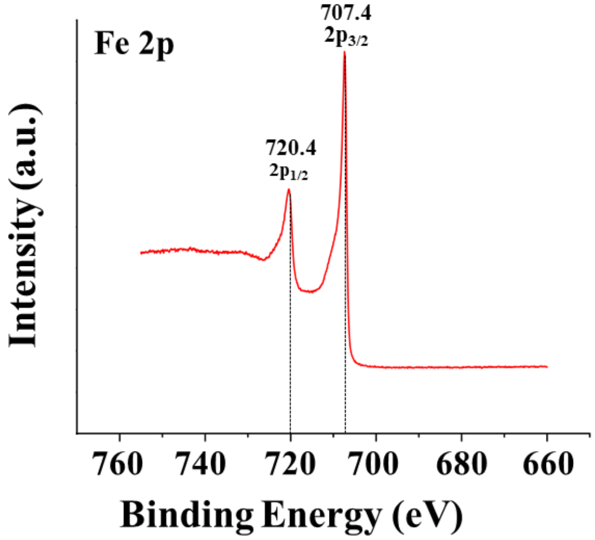

fig. S10. Fe 2p and C 1s XPS characterization of ICNPs after 5 cycles of magnetocatalytic hydrodeoxygenation of methyl benzoate (**1**) to toluene (**1a**).

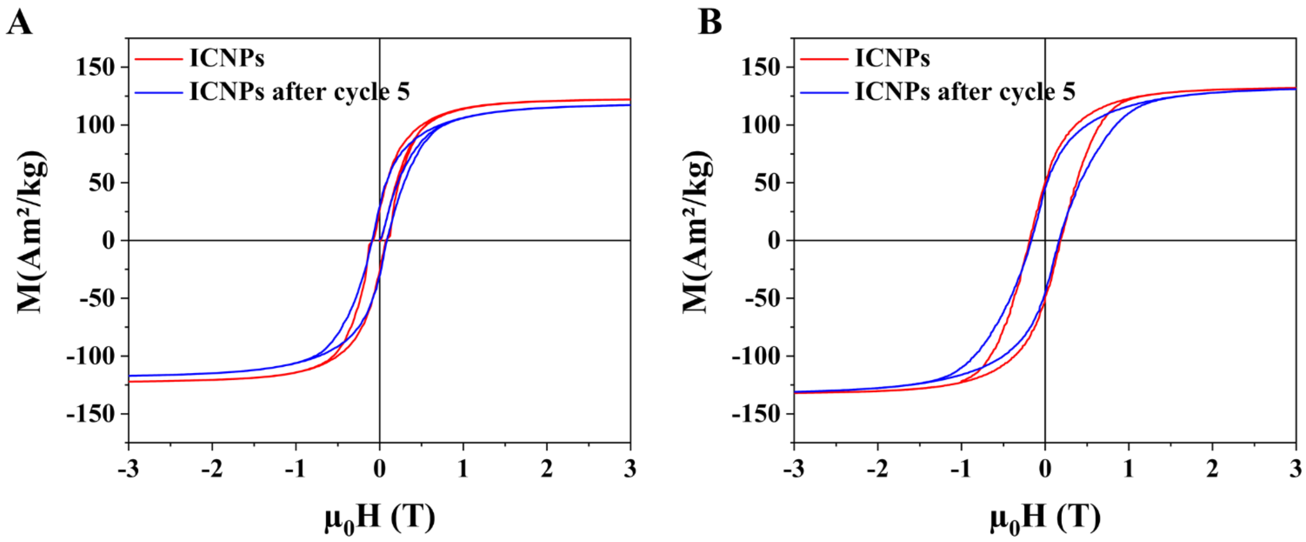

fig. S11. VSM characterization of ICNPs before and after 5 cycles. (**A**) at 300 K and (**B**) at 5 K of ICNPs before (red) and ICNPs after five cycles (blue) of magnetocatalytic hydrodeoxygenation of methyl benzoate (**1**) to toluene (**1a**).

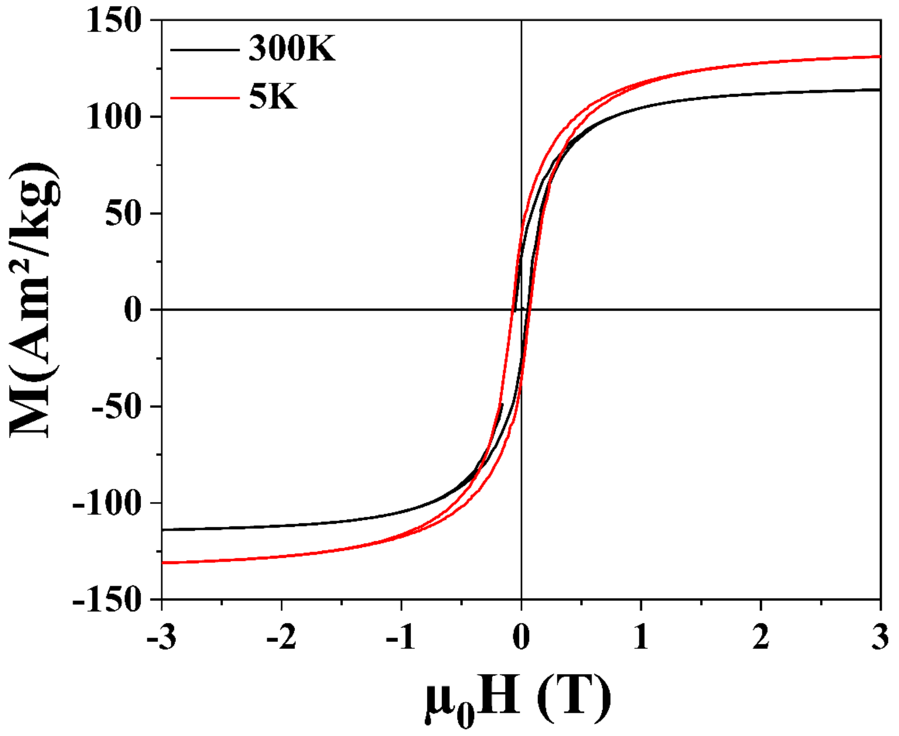

fig. S12. VSM characterization at 300 K and 5 K of ICNPs after five cycles of conventional heating hydrodeoxygenation of methyl benzoate (**1**) to toluene (**1a**).

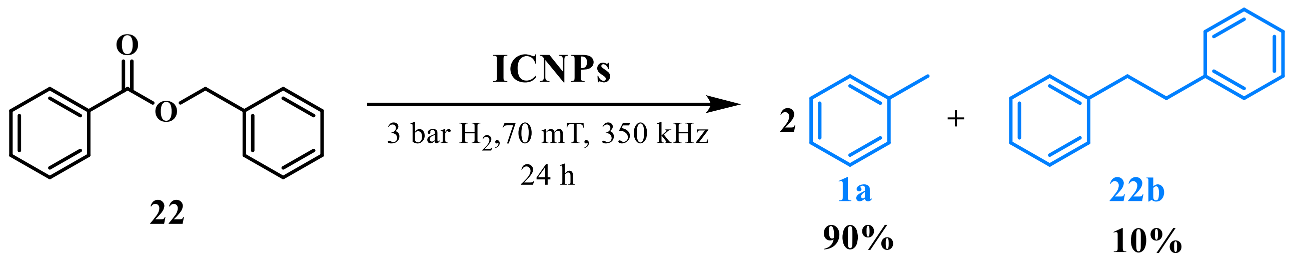

scheme S1. 500 mg-scale hydrodeoxygenation of substrate **22** (2.63 mmol, 21 eq w.r.t. total Fe, 350 eq w.r.t. Fe available at the ICNPs' surface) using ICNPs (10 mg, 0.125 mmol Fe, of which ca. 6% is localized at the NPs surface) under 3 bar H_2, 24 h, ACMF (70 mT, 350 kHz).

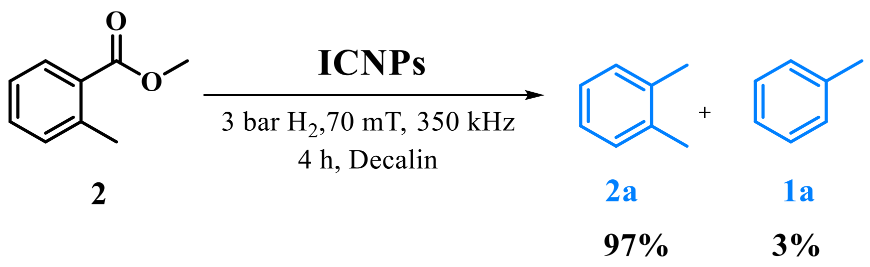

scheme S2. Product distribution for the hydrodeoxygenation of methyl 2-methylbenzoate (**2**).

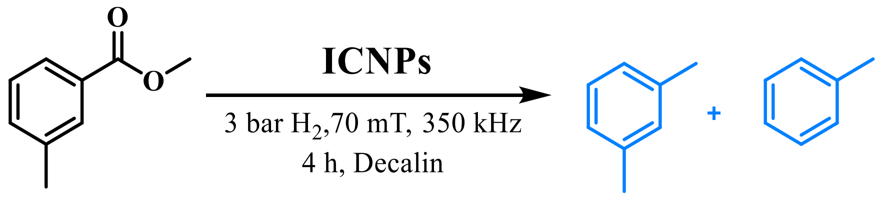

3 **3a** **1a**

98% **2%**

scheme S3. Product distribution for the hydrodeoxygenation of methyl 3-methylbenzoate (**3**).

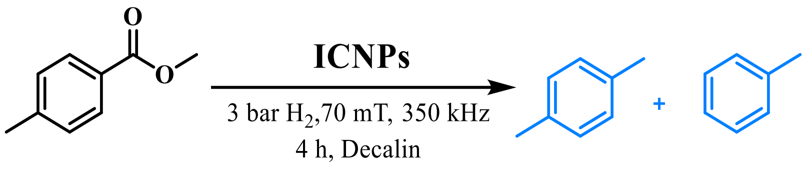

4 **4a** **1a**

98% **2%**

scheme S4. Product distribution for the hydrodeoxygenation of methyl 4-methylbenzoate (**4**).

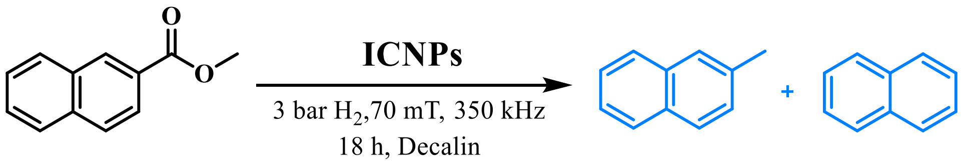

5 **5a** **5b**

92% **8%**

scheme S5. Product distribution for the hydrodeoxygenation of methyl 2-naphthoate (**5**).

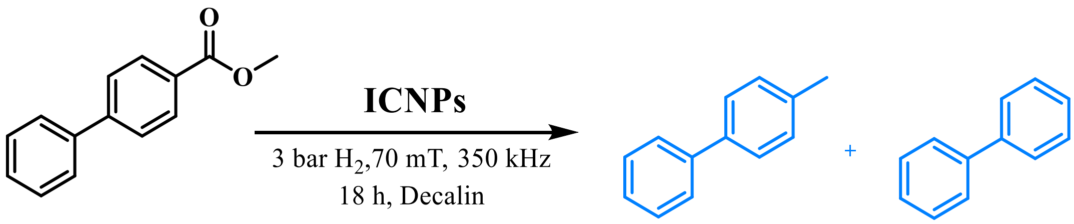

6 **6a** **6b**

93% **8%**

scheme S6. Product distribution for the hydrodeoxygenation of methyl [1,1'-biphenyl]-4-carboxylate (**6**).

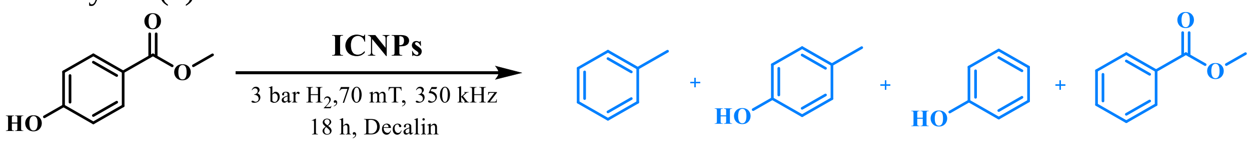

7 **1a** **7a** **7b** **1**

64% **20%** **7%** **6%**

scheme S7. Product distribution for the hydrodeoxygenation of methyl 4-hydroxybenzoate (**7**).

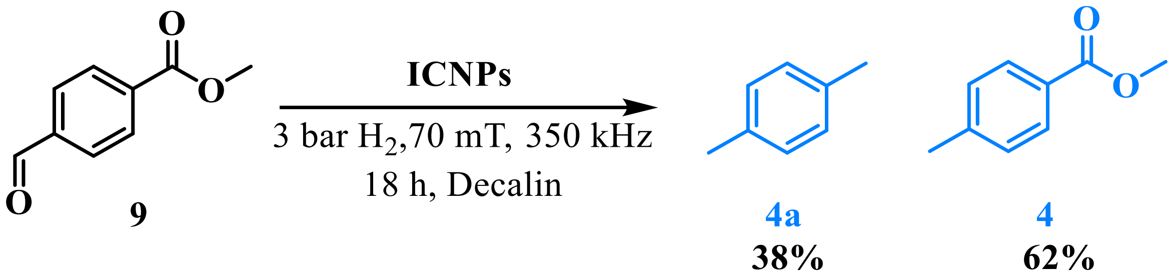

scheme S8. Product distribution for the hydrodeoxygenation of methyl 4-formylbenzoate (**9**).

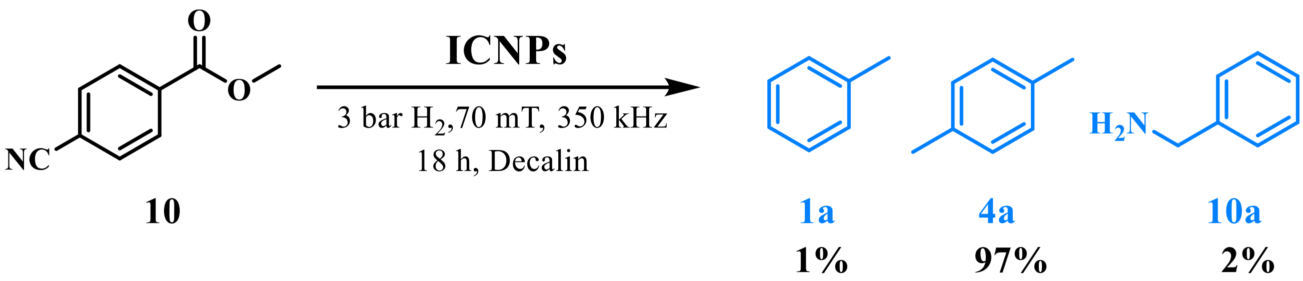

scheme S9. Product distribution for the hydrodeoxygenation of methyl 4-cyanobenzoate (**10**).

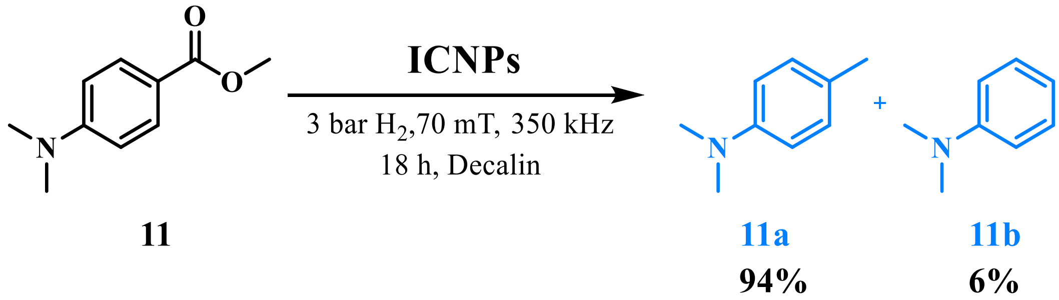

scheme S10. Product distribution for the hydrodeoxygenation of methyl 4-(dimethylamino)benzoate (**11**).

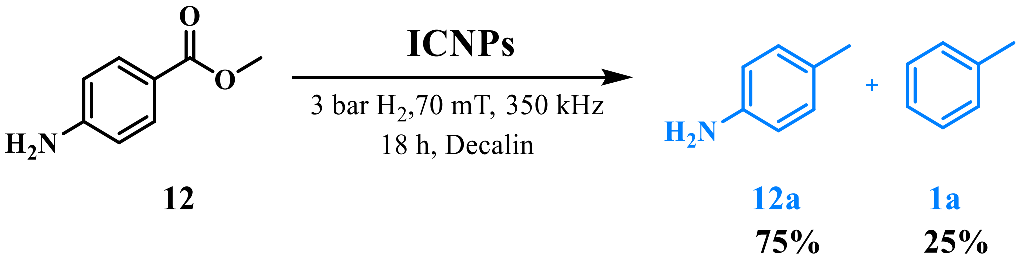

scheme S11. Product distribution for the hydrodeoxygenation of methyl 4-aminobenzoate (**12**).

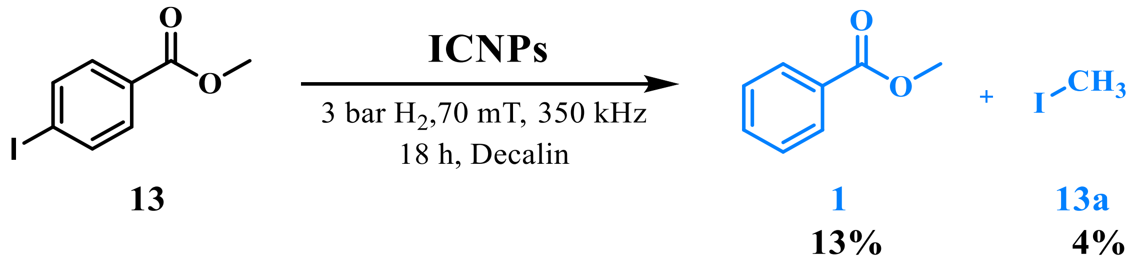

scheme S12. Product distribution for the hydrodeoxygenation of methyl 4-iodobenzoate (**13**).

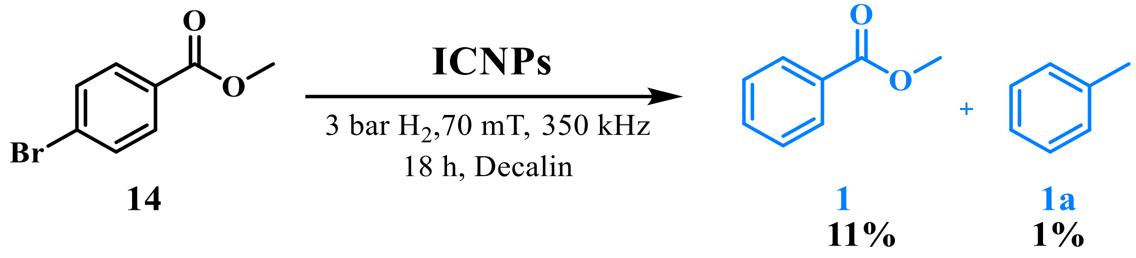

scheme S13. Product distribution for the hydrodeoxygenation of methyl 4-bromobenzoate (**14**).

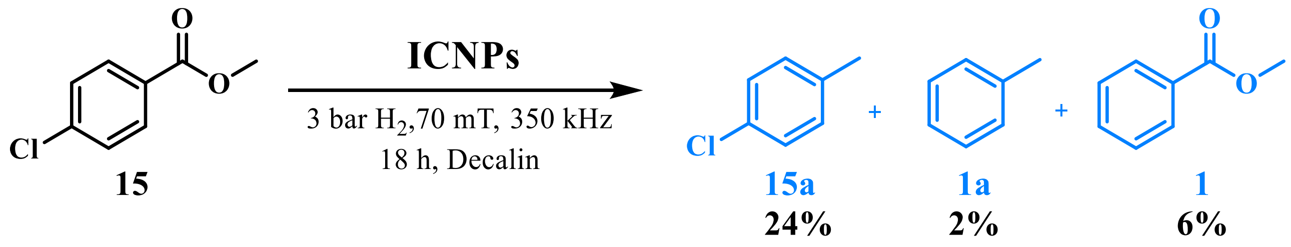

scheme S14. Product distribution for the hydrodeoxygenation of methyl 4-chlorobenzoate (**15**).

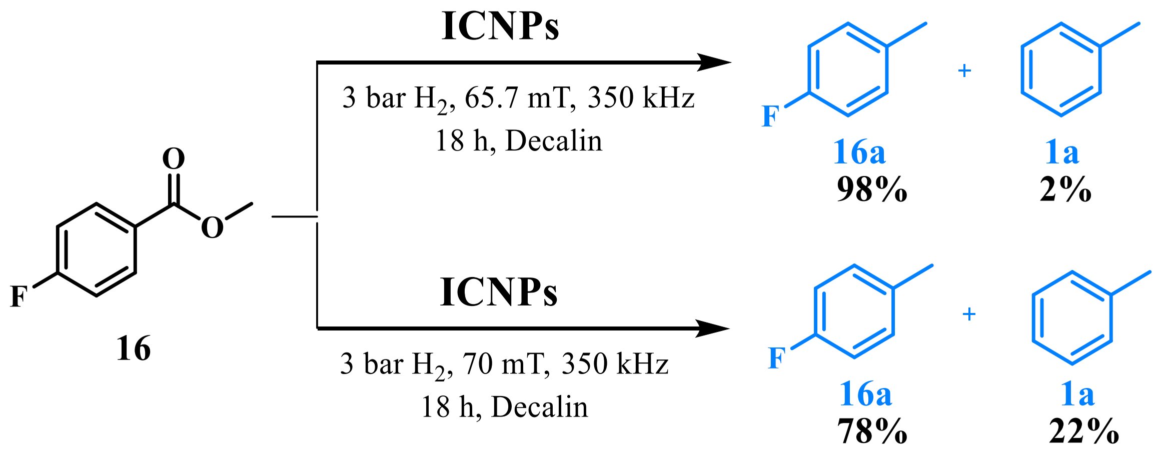

scheme S15. Product distribution for the hydrodeoxygenation of methyl 4-fluorobenzoate (**16**).

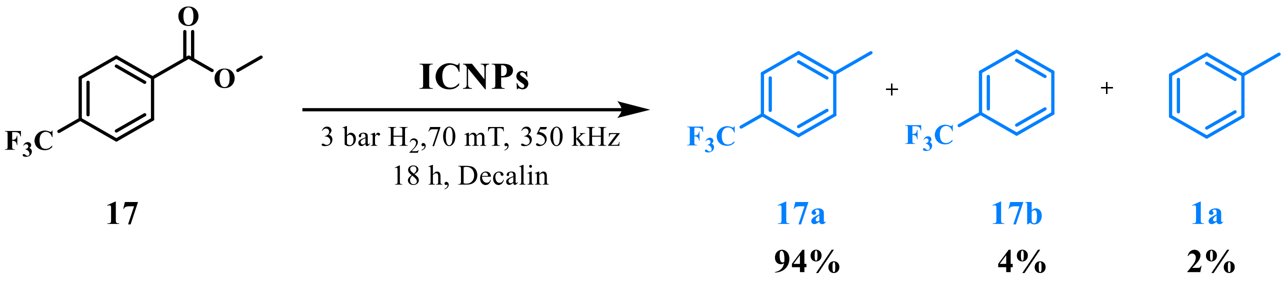

scheme S16. Product distribution for the hydrodeoxygenation of methyl 4-(trifluoromethyl)benzoate (**17**).

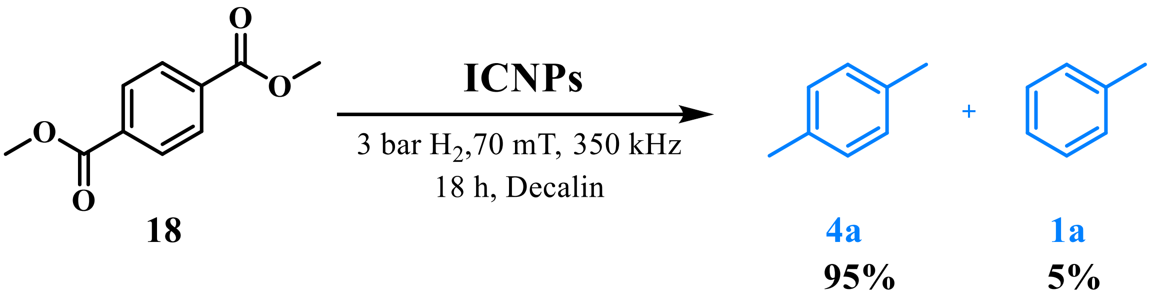

scheme S17. Product distribution for the hydrodeoxygenation of dimethyl terephthalate (**18**).

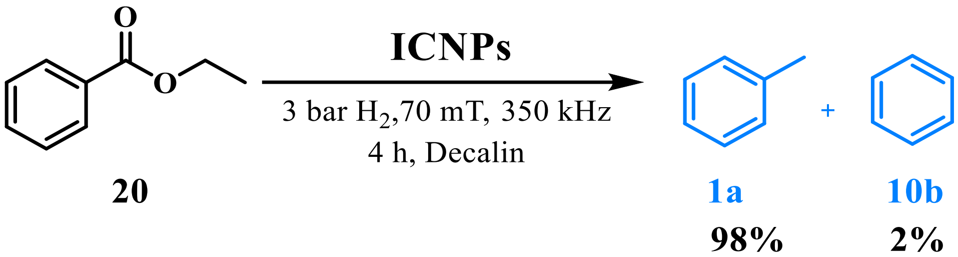

scheme S18. Product distribution for the hydrodeoxygenation of ethyl benzoate (**20**).

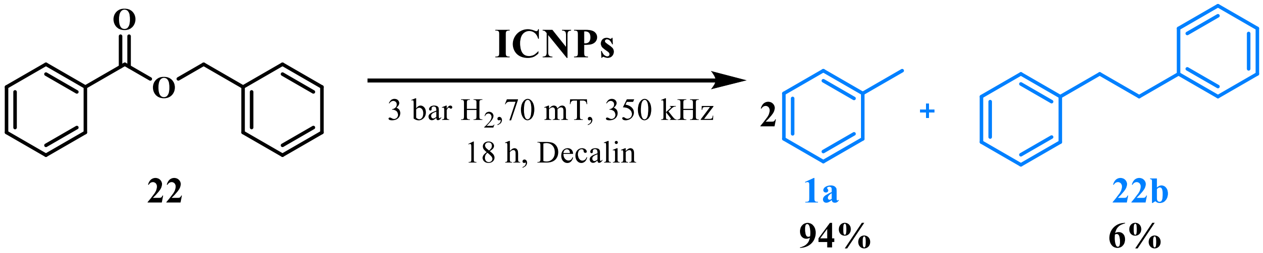

scheme S19. Product distribution for the hydrodeoxygenation of benzyl benzoate (**22**).

table S1. VSM Analysis of Fe(0) nanoparticles.

	**Fe(0), 300 K**	**Fe(0), 5 K**	**ICNPs, 300 K**	**ICNPs, 5 K**
**M_s [Am^2/kg]**	156	172	122	132
**M_r [Am^2/kg]**	10	41	28	50
**M_r/M_s [%]**	6.4	23.8	23.0	37.9
**$\mu_0 H_c$ [mT]**	10	88	80	180

table S2. Variation of solvents for the magnetocatalytic hydrodeoxygenation of **1** with ICNPs (otherwise standard conditions).

**Entry**	**Solvent/Solid**	**Boiling/Melting Point [°C]**	**Global Temperature [°C]**	**Boiling/Melting upon magnetic induction?**
1	Heptane	98	201	Yes
2	Mesitylene	165	205	Yes
3	Decalin	186	198	Yes
4	Dodecane	216	197	Yes
5[a]	Propylene carbonate	242	202	Yes
6	Tetradecane	254	199	Yes
7	Sulfolane	285	204	Yes
8	Hexadecane	287	202	Yes
9	Tetraethylene glycol	327	201	Yes
10	$Mg(OH)_2$	350	199	No

Local temperature at solvent/ICNPs interface estimated from local boiling/gas bubble formation of respective solvent. For entry 10, 50 mg of $Mg(OH)_2$ were used. Global temperatures were determined by IR camera. [a]=experiment was conducted for only 30 min as pressure increases to +2 bar due to decomposition of propylene carbonate to CO_2.

table S3. Variation of ACMF amplitude for the magnetocatalytic hydrodeoxygenation of **1** with ICNPs (otherwise standard conditions).

Entry	$\mu_0 H_{max}$ [mT]	Global Temperature [°C]	Reaction Time [h]	Selectivity [%]	Yield 1a [%]
1	50	121	4	$\geq$99	30
2	55.6	135	4	$\geq$99	45
3	65.7	150	4	$\geq$99	75
4	70	200	4	$\geq$99	$\geq$99
5	74	225	4	$\geq$99	$\geq$99

Reaction conditions: methyl benzoate **1** (44.9 mg, 0.33 mmol), ICNPs (10 mg, 0.125 mmol of Fe), decalin (0.5 mL), H_2 (3 bar), magnetic field (350 kHz). The product selectivity is >99%. Product yields determined by GC-FID using tetradecane as the internal standard. Global temperature determined by IR camera.

table S4. Conversion of benzoic acid with ICNPs under standard conditions.

Substrate	Solvent	Select.(%)	Yield 1a (%)
Benzoic acid	decalin	0	0

Reaction conditions: benzoic acid (39.7 mg, 0.33 mmol), ICNPs (10 mg, 0.125 mmol of Fe), decalin (0.5 mL), H_2 (3 bar), magnetic field (70 mT, 350 kHz), 1 h. The product selectivity is >99%. Product yields determined by GC-FID using tetradecane as the internal standard.

table S5. Impact of stirring in the HDO of **1** under conventional heating at 200 °C.

Entry	Stirring [rpm]	Temperature [°C]	Reaction Time [h]	Selectivity [%]	Yield 1a [%]
1	0	200	4	>99	6
2	100	200	4	>99	3
3	700	200	4	>99	5

Reaction conditions: methyl benzoate **1** (44.9 mg, 0.33 mmol), ICNPs (10 mg, 0.125 mmol of Fe), decalin (0.5 mL), H_2 (3 bar), 200 °C. The product selectivity is >99%. Product yields determined by GC-FID using tetradecane as the internal standard.

table S6. Summary of the characterization of ICNPs after five cycles of magnetocatalytic hydrodeoxygenation of methyl benzoate (**1**) to toluene (**1a**).

	**fresh ICNPs**			**ICNPs 5 cycles**		
**NPs size [nm]**	12.4 ± 1.1			12.3 ± 1.7		
**^{57}Fe Mössbauer Analysis: Content [%]**	Paramagnetic species = 4%<br>$Fe_{2.2}C$=76%<br>Fe_5C_2=20%			Paramagnetic species = 3%<br>$Fe_{2.2}C$=68%<br>Fe_5C_2=28%		
**XPS analysis: Binding Energy [eV]**	Fe 2p	$2p_{1/2}$ = 720.3<br>$2p_{3/2}$ = 707.6		Fe 2p	$2p_{1/2}$=720.4<br>$2p_{3/2}$=707.4	
	C 1s	Graphite, organics = 288.8<br>1 s = 285.5<br>1 s = 283.3		C 1s	Graphite, organics = 288.8<br>1 s = 285.2<br>1 s = 283.3	
**VSM Analysis**	M_s [A m^2/kg]	M_s (300 K)= 122	M_s (5 K) = 132	M_s [A m^2/kg]	M_s (300 K)= 117	M_s (5 K) = 131
	μ_0H_c [mT]	H_c (300 K) = 80	H_c (5 K) = 180	μ_0H_c [mT]	H_c (300 K) = 88	H_c (5 K) = 166

table S7. ICP-MS analysis of the Fe content in reaction solutions (1 mL) after cycles of 2 h under standard (magnetocatalytic) conditions (refers to Figure 5).

**Reaction cycle**	**Fe concentration [μg/L]**
1	1.5 ± 3.6
2	2.9 ± 3.7
3	3.1 ± 10.1
4	3.9 ± 5.2
5	3.3 ± 4.1

table S8. Elemental analysis of ICNPs by inductively coupled plasma mass spectroscopy (ICP-MS).

Catalyst	Fe content (wt%)
Fresh ICNPs	83.2
ICNPs after cycle 3	88.5

table S9. ICP-MS analysis of the Fe content in reaction solutions (1 mL) after cycles of 2 h under conventional heating at 350 °C (refers to Figure 5).

Reaction cycle	Fe concentration [μg/L]
1	5.1 ± 3.8
2	3.4 ± 23.1
3	2.9 ± 5.2
4	1.8 ± 2.9
5	7.0 ± 3.7

Isolated Yields

*Hydrodeoxygenation of dimethyl terephthalate **18***

General procedure: ICNPs (10.0 mg) and dimethyl terephthalate (**18**, 64.1 mg, 0.33 mmol) were added in a Fisher-Porter bottle (without solvent) inside the glovebox, then sealed and pressurized with H_2 (3 bar). The Fisher-Porter bottle was placed in the coil under an alternating magnetic field (70 mT, 350 kHz) for 24 h. After the reaction was finished, the catalyst was removed by magnetic separation. The desired product was washed with acetone and distilled for purification. *p*-xylene yield = 89 % (31.1 mg).

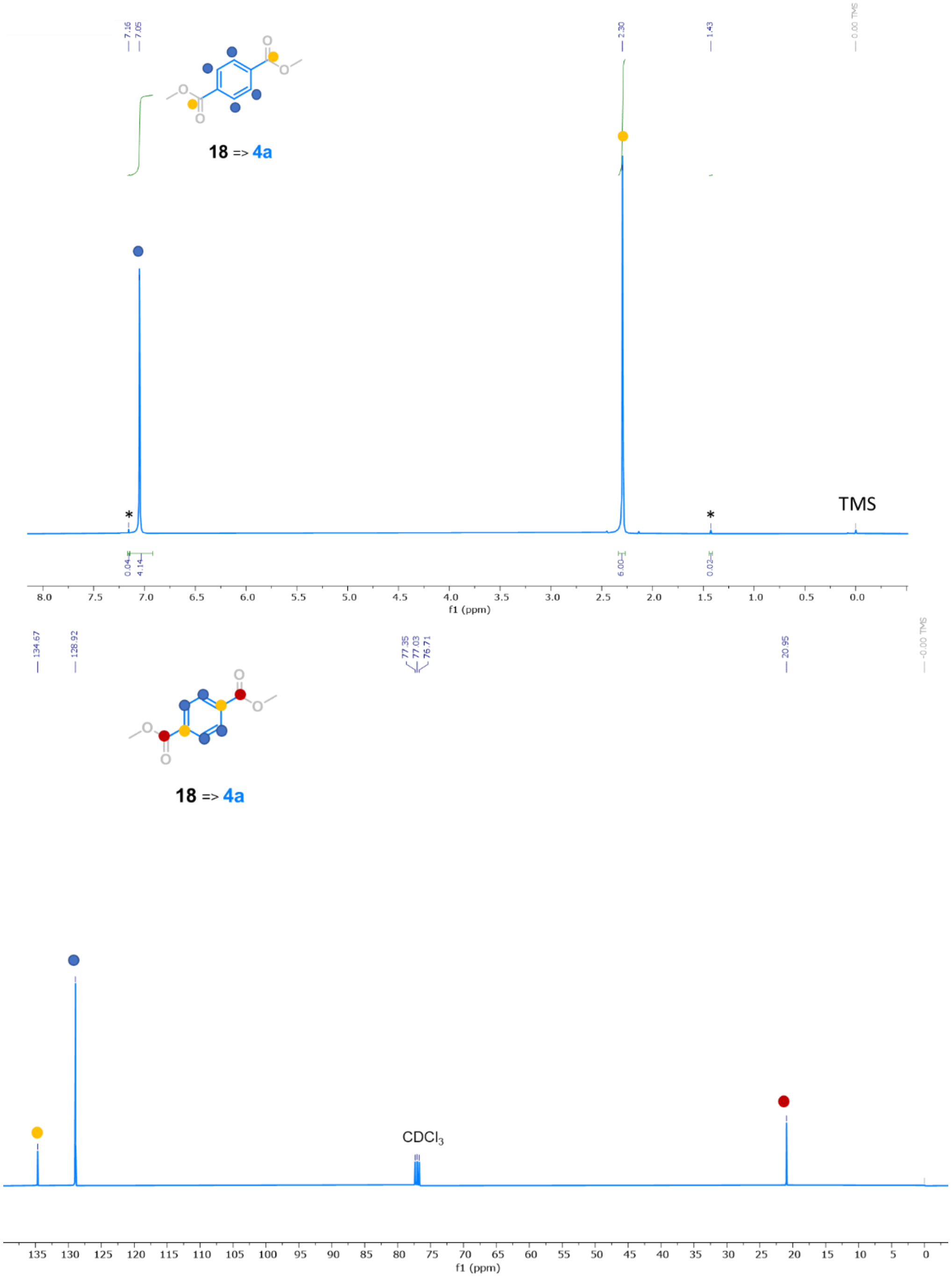

fig. S13. ^{1}H and ^{13}C NMR spectra of isolated **4a**. * = solvent impurities.

Hydrodeoxygenation of benzyl benzoate ***22***

General procedure: ICNPs (10.0 mg) and benzyl benzoate **21** (557.2 mg, 2.63 mmol) were added in a Fisher-Porter bottle (without solvent) inside the glovebox, then sealed and pressurized with H_2 (3 bar). The Fisher-Porter bottle was placed in the coil under an alternating magnetic field (70 mT, 350 kHz) for 24 h. After the reaction was finished, the catalyst was removed by magnetic separation. The desired product was washed with acetone and distilled for purification. Toluene yield = 90 % (438.9 mg).

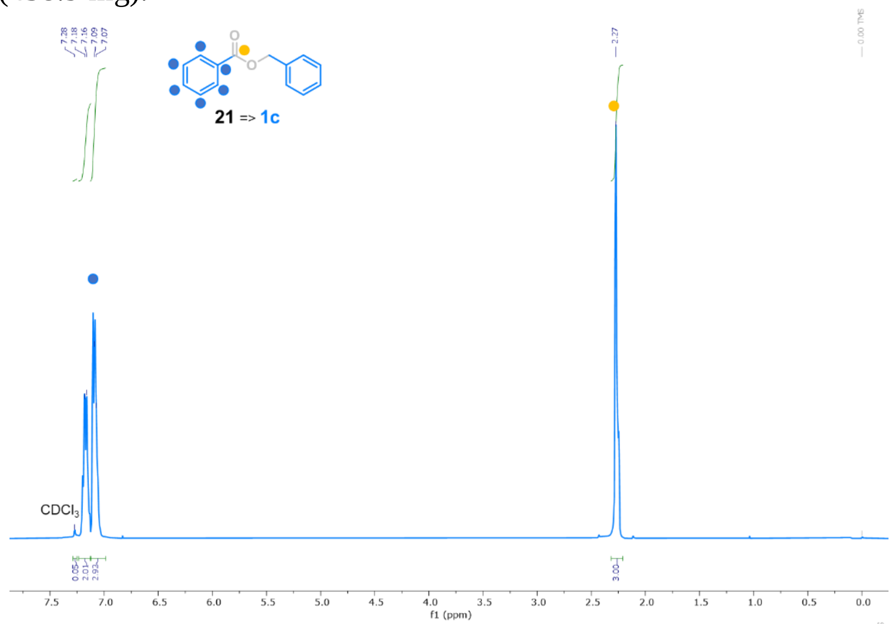

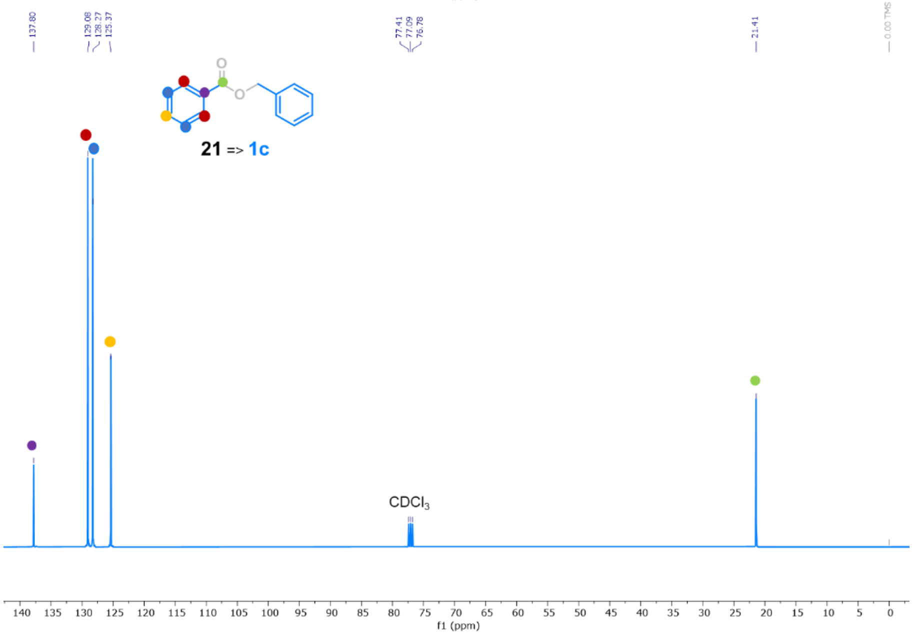

fig. S14. ^{1}H and ^{13}C NMR spectra of isolated **1a.**

PET Recycling

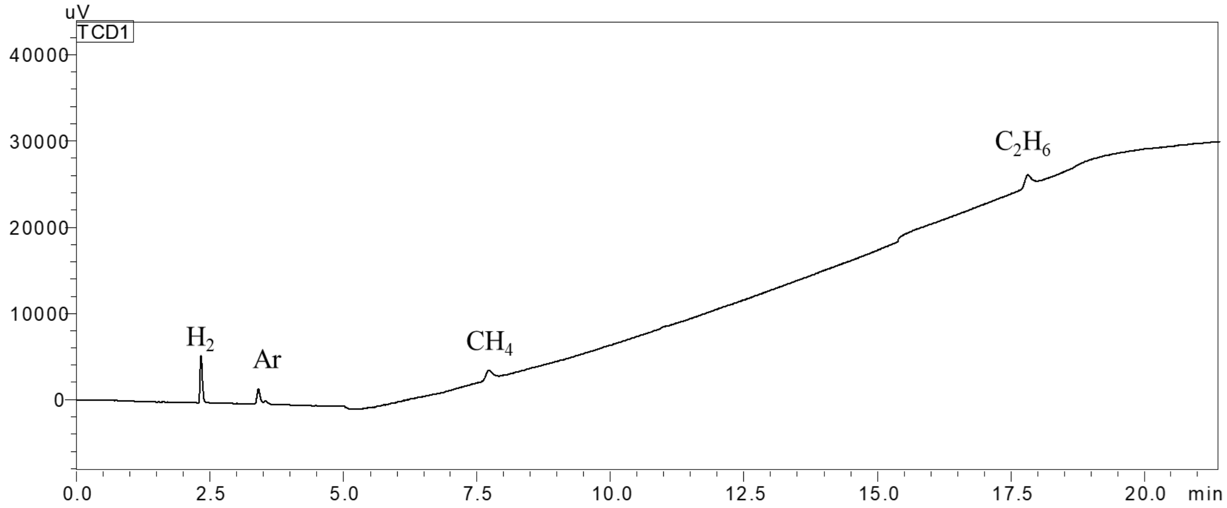

fig. S15. GC-Headspace, gas phase analysis after 24 h reaction with PET (0.33 mmol) under standard conditions, 24 h.

References

1 J. Pritchard, G. A. Filonenko, R. Van Putten, E. J. Hensen, E. A. Pidko, Heterogeneous and homogeneous catalysis for the hydrogenation of carboxylic acid derivatives: history, advances and future directions. *Chem. Soc. Rev.* **44**, 3808-3833 (2015).

2 K. Lee, Y. Jing, Y. Wang, N. Yan, A unified view on catalytic conversion of biomass and waste plastics. *Nature Rev. Chem.* **6**, 635-652 (2022).

3 T. V. Choudhary, C. B. Philips, Renewable fuels via catalytic hydrodeoxygenation. *Appl. Catal. A: General* **397**, 1-12 (2011).

4 J. Seyden-Penne, Reductions by the alumino-and borohydrides in organic synthesis. (John Wiley & Sons, 1997).

5 J. Kollonitsch, O. Fuchs, Preparation of aluminum borohydride and its applications in organic reductions. *Nature* **176**, 1081 (1955).

6 C. J. Barger, A. Motta, V. L. Weidner, T. L. Lohr, T. J. Marks, $La[N(SiMe_3)_2]_3$-catalyzed ester reductions with pinacolborane: Scope and mechanism of ester cleavage. ACS *Catal.* **9**, 9015-9024 (2019).

7 A. Cook, S. Prakash, Y.-L. Zheng, S. G. Newman, Exhaustive reduction of esters enabled by nickel catalysis. *JACS* **142**, 8109-8115 (2020).

8 B. Han, C. Ren, M. Jiang, L. Wu, Titanium-Catalyzed Exhaustive Reduction of Oxo-Chemicals. *Angew. Chem. Int. Ed.* **61**, e202209232 (2022).

9 M. K. Sahu, S. Pattanaik, C. Gunanathan, Cobalt-catalyzed reduction of esters to alkanes. *Chem. Comm.* **61**, 1661-1664 (2025).

10 Y. Li, M. Wang, X. Liu, C. Hu, D. Xiao, D. Ma, Catalytic Transformation of PET and CO_2 into High-Value Chemicals. *Angew. Chem. Int. Ed.* **61**, e202117205 (2022).

11 R. Helmer, S. S. Borkar, A. Li, F. Mahnaz, J. Vito, M. Bishop, A. Iftakher, M. M. Faruque Hasan, S. Rangarajan, M. Shetty, Tandem Methanolysis and Catalytic Transfer Hydrogenolysis of Polyethylene Terephthalate to p-Xylene Over Cu/ZnZrOx Catalysts. *Angew. Chem. Int. Ed.* **64**, e202416384 (2025).

12 A. Bordet, W. Leitner, B. Chaudret, Magnetically Induced Catalysis: Definition, Advances and Potential. *Angew. Chem. Int. Ed.,* e202424151 (2025).

13 J. Mazarío, S. Ghosh, V. Varela-Izquierdo, L. M. Martínez-Prieto, B. Chaudret, Magnetic Nanoparticles and Radio Frequency Induction: From Specific Heating to Magnetically Induced Catalysis. *ChemCatChem* **17**, e202400683 (2025).

14 S. Ceylan, C. Friese, C. Lammel, K. Mazac, A. Kirschning, Inductive heating for organic synthesis by using functionalized magnetic nanoparticles inside microreactors. *Angew. Chem. Int. Ed*. **47**, 8950-8953 (2008).

15 A. Bordet, L. M. Lacroix, P. F. Fazzini, J. Carrey, K. Soulantica, B. Chaudret, Magnetically induced continuous CO_2 hydrogenation using composite iron carbide nanoparticles of exceptionally high heating power. *Angew. Chem. Int. Ed*. **55**, 15894-15898 (2016).

16 A. Adogwa, E. Chukwu, A. Malaj, V. R. Punyapu, O. Chamness, N. Glisson, B. Bruce, S. Lee, M. J. Zachman, D. A. Bruce, R. B. Getman, D. T. Mefford, M. Yang, Catalytic reaction triggered by magnetic induction heating mechanistically distinguishes itself from the standard thermal reaction. *ACS Catal.* **14**, 4008-4017 (2024).

17 H. Kreissl, J. Jin, S. H. Lin, D. Schüette, S. Störtte, N. Levin, B. Chaudret, A. Vorholt, A. Bordet, W. Leitner, Commercial $Cu_2Cr_2O_5$ decorated with iron carbide nanoparticles as a multifunctional catalyst for magnetically induced continuous-flow hydrogenation of aromatic ketones. *Angew. Chem. Int. Ed*. **60**, 26639-26646 (2021).

18 S. H. Lin, S. Ahmedi, C. Campalani, A. Kretschmer, Y. Kayser, L. Kang, S. DeBeer, W. Leitner, A. Bordet, Low Pressure Amide Hydrogenation Enabled by Magnetocatalysis. *Nat. Comm*., *In Press* (2025).

19 J. Chastain, R. C. King Jr, Handbook of X-ray photoelectron spectroscopy. Perkin-Elmer Corporation 40, 25 (1992).

20 Y. Hirotsu, S. Nagakura, Crystal Structure and Morphology of the Carbide Precipitated from Martensitic High Carbon Steel During the First Stage of Tempering. *Acta Metall*., **20**, 645-655 (1972).

21 M. Žula, M. Grilc, B. Likozar, Hydrocracking, hydrogenation and hydro-deoxygenation of fatty acids, esters and glycerides: Mechanisms, kinetics and transport phenomena. *J. Chem. Eng.* **444**, 136564 (2022).

22 P. R. Jones, Ch. A. Chuang, T. Sun, T. Zhao, K. Fezza, J.C. Takase, D. Singh, N. A. Patankar, High-speed X-ray imaging of the Leidenfrost collapse. *Sci. Rep.* **9**, 1598 (2019).

23 E. da Silva Nunes, W. R. Viali, S. W. da Silva, J. A. H. Coaquira, V. K. Garg, A. C. de Oliveira, P. C. Morais, M. J. Júnior, Characterization of tetraethylene glycol passivated iron nanoparticles. *Appl. Surf. Sci.* **315**, 337-345 (2014).

24 A. Bordet, W. Leitner, Adaptive catalytic systems for chemical energy conversion. *Angew. Chem. Int. Ed*. **62**, e202301956 (2023).

25 J. P. Noble, S. J. Bending, A. K. Hill, Radiofrequency Induction Heating for Green Chemicals Manufacture: A Systematic Model of Energy Losses and a Scale-Up Case-Study. *ACS Engineering Au* **4**, 450-463 (2024).

26 P. Wang, F.-K. Chiang, J. Chai, A. I. Dugulan, J. Dong, W. Chen, R. J. P. Broos, B. Feng, Y. Song, Y. Lv, Q. Lin, R. Wang, I. A. W. Filot, Z. Men, E. J. M. Hensen, Efficient conversion of syngas to linear α-olefins by phase-pure χ-Fe_5C_2. *Nature* **635**, 102-107 (2024).

27 Y. Hirayama, A. Miura, M. Hirayama, H. Nakamura, K. Fujita, H. Kageyama, S. Yamaguchi, T. Mizugaki, T. Mitsudome. One-Step Low-Temperature Synthesis of Metastable ε-Iron Carbide Nanoparticles with Unique Catalytic Properties Beyond Conventional Iron Catalysts. *Small.* **21**, 2412217 (2025).

28 J. M. Asensio, A. B. Miguel, P. F. Fazzini, P. W. Van Leeuwen, B. Chaudret, Hydrodeoxygenation using magnetic induction: high-temperature heterogeneous catalysis in solution. *Angew. Chem.* **131**, 11428-11432 (2019).

29 S. H. Lin, W. Hetaba, B. Chaudret, W. Leitner, A. Bordet, Copper-Decorated Iron Carbide Nanoparticles Heated by Magnetic Induction as Adaptive Multifunctional Catalysts for the Selective Hydrodeoxygenation of Aldehydes. *Adv. Energy Mater.* **12**, 2201783 (2022).

30 Hong, Y., Zhang, H., Sun, J., Ayman, K. M., Hensley, A. J. R., Gu, M., Engelhard, M. H., McEwen, J. S. & Wang, Y. Synergistic catalysis between Pd and Fe in gas phase hydrodeoxygenation of m-cresol. *ACS Catal.* **4**, 3335-3345 (2014).

31 Gupta, U., Yadav, M., Saini, B., Krishnapriya, R. & Sharma, R. K. Fe_xNi_y/SiO_2-Al_2O_3 catalyzed hydrodeoxygenation of biorenewable platform molecules. *Fuel* **360**, 130588 (2024).

32 J. J. Retief, Powder diffraction data and Rietveld refinement of Hägg-carbide, χ-Fe_5C_2. *Powder Diffr.*, **14**, 130-132 (1999).

33 F. Baur, D. Beattie, D. Beer, D. Bentley, M. Bradley, I. Bruce, S. J. Charlton, B. Cuenoud, R. Ernst, R. A. Fairhurst, B. Faller, D. Farr, T. Keller, J. R. Fozard, J. Fullerton, S. Garman, J. Hatto, C. Hayden, H. He, C. Howes, D. Janus, Z. Jiang, C. Lewis, F. Loeuillet-Ritzler, H. Moser, J. Reilly, A. Steward, D. Skyes, L. Tedaldi, A. Trifilieff, M. Tweed, S. Watson, E. Wissler, D. Wyss, The Identification of Indacaterol as an Ultralong-Acting Inhaled β_2-Adrenoceptor Agonist. *J. Med. Chem.* **53**, 3675-3684 (2010).

34 D. Wu, Y. Duan, K. Liang, H. Yin, F.-X. Chen, AIBN-initiated direct thiocyanation of benzylic sp^3 C–H with *N*-thiocyanatosaccharin. *Chem. Commun.* **57**, 9938-9941 (2021).

35 Z. Alassad, A. Aboraed, M. S. Mizrachi, M. H. Pérez-Temprano, A. Milo, Metal-Free Multicomponent Strategy for Amidine Synthesis. *J. Am. Chem. Soc.* **144**, 20672-20679 (2022).

36 F. Diness, D. P. Fairlie, Catalyst-Free N-Arylation Using Unactivated Fluorobenzenes. *Angew. Chem. Int. Ed.* **51**, 8012-8016 (2012).

37 J. Zhang, G. Lu, J. Xu, H. Sun, Q. Shen, Nickel-Catalyzed Reductive Cross-Coupling of Benzyl Chlorides with Aryl Chlorides/Fluorides: A One-Pot Synthesis of Diarylmethanes. *Org. Let.* **18**, 2860-2863 (2016).

38 C. Shi, E. C. Quinn, W. T. Diment, E. Y.-X. Chen, Recyclable and (Bio)degradable Polyesters in a Circular Plastics Economy. *Chem. Rev.* **124**, 4393-4478 (2024).

39 D. L. Broere, I. Čorić, A. Brosnahan, P. L. Holland, Quantitation of the THF Content in $Fe[N(SiMe_3)_2]_2 \cdot$ x THF. *Inorg. Chem.* **56**, 3140-3143 (2017).

40 C. P. Harmer, S. Kamali, O. I. Lebedev, S. J. Lee, R. A. Ribeiro, P. C. Canfield, K. Kovnir, Pseudo-polymorphism in layered FeS intercalates: A competition between charged and neutral guest species. *Chem. Mater.* **34**, 5397-5408 (2022).

41 M. E. Fleet. The structure of Magnetite. *Acta Cryst.* **B37**, 917-920 (1981).
